# Novel and potent inhibitors targeting DHODH are broad-spectrum antivirals against RNA viruses including newly-emerged coronavirus SARS-CoV-2

**DOI:** 10.1007/s13238-020-00768-w

**Published:** 2020-08-04

**Authors:** Rui Xiong, Leike Zhang, Shiliang Li, Yuan Sun, Minyi Ding, Yong Wang, Yongliang Zhao, Yan Wu, Weijuan Shang, Xiaming Jiang, Jiwei Shan, Zihao Shen, Yi Tong, Liuxin Xu, Yu Chen, Yingle Liu, Gang Zou, Dimitri Lavillete, Zhenjiang Zhao, Rui Wang, Lili Zhu, Gengfu Xiao, Ke Lan, Honglin Li, Ke Xu

**Affiliations:** 1 State Key Laboratory of Virology, College of Life Sciences, Wuhan University, 430072 Wuhan, China; 2 Shanghai Key Laboratory of New Drug Design, State Key Laboratory of Bioreactor Engineering, School of Pharmacy, East China University of Science and Technology, 200237 Shanghai, China; 3 State Key Laboratory of Virology, Wuhan Institute of Virology, Center for Biosafety Mega-Science, Chinese Academy of Sciences, 430071 Wuhan, China; 4 CAS Key Laboratory of Molecular Virology and Immunology, Institut Pasteur of Shanghai, University of Chinese Academy of Sciences, Chinese Academy of Sciences, 200031 Shanghai, China

**Keywords:** *de novo* pyrimidine biosynthesis, DHODH inhibitors, SARS-CoV-2, influenza viruses, virus replication, immuno-regulation

## Abstract

Emerging and re-emerging RNA viruses occasionally cause epidemics and pandemics worldwide, such as the on-going outbreak of the novel coronavirus SARS-CoV-2. Herein, we identified two potent inhibitors of human DHODH, S312 and S416, with favorable drug-likeness and pharmacokinetic profiles, which all showed broad-spectrum antiviral effects against various RNA viruses, including influenza A virus, Zika virus, Ebola virus, and particularly against SARS-CoV-2. Notably, S416 is reported to be the most potent inhibitor so far with an EC_50_ of 17 nmol/L and an SI value of 10,505.88 in infected cells. Our results are the first to validate that DHODH is an attractive host target through high antiviral efficacy *in vivo* and low virus replication in DHODH knock-out cells. This work demonstrates that both S312/S416 and old drugs (Leflunomide/Teriflunomide) with dual actions of antiviral and immuno-regulation may have clinical potentials to cure SARS-CoV-2 or other RNA viruses circulating worldwide, no matter such viruses are mutated or not.

## Introduction

Acute viral infections, such as influenza virus, SARS-CoV, MERS-CoV, Ebola virus, Zika virus, and the very recent SARS-CoV-2 are an increasing and probably lasting global threat (Gao, 2018). Existing direct-acting antiviral (DAA) drugs cannot be applied immediately to new viruses because of virus-specificity, and the development of new DAA drugs from the beginning is not timely for outbreaks. Broad-spectrum antivirals (BSA) are clinically needed for the effective control of emerging and re-emerging viral infectious diseases. However, although great efforts have been made by the research community to discover therapeutic antiviral agents for coping with such emergencies, yet specific and effective drugs or vaccines with low toxicity have been rarely reported (Ianevski et al., 2019). Up to now, unfortunately, there are still no effective drugs for the cure of individuals who are infected with the novel coronavirus, such as SARS-CoV-2, in which an unprecedented outbreak of this virus had occurred in December 2019. This coronavirus was firstly identified in early January 2020 (Chen et al., 2020; Wu et al., 2020; Zhou et al., 2020) and now has quickly spread throughout the globe, infected more than 10 million individuals and taken the lives of 512, 842 among them as of July 3, 2020.

Discovery of nucleoside or nucleotide analogs and host-targeting antivirals (HTAs) are two main strategies for developing BSA (Min and Subbarao, 2010; Jordheim et al., 2013; Jordan et al., 2018). With the former drug class usually causing drug resistance and toxicity, the discovery of HTAs has attracted much attention (Adalja and Inglesby, 2019). Several independent studies searching for HTAs collectively end up to compounds targeting the host’s pyrimidine synthesis pathway to inhibit virus infections, which indicates that the replication of viruses is widely dependent on the host pyrimidine synthesis (Zeng et al., 2005; Qing et al., 2010; Hoffmann et al., 2011; Das et al., 2013; Lucas-Hourani et al., 2013, 2017; Marschall et al., 2013; Raveh et al., 2013; Chung et al., 2016; Grandin et al., 2016; Cheung et al., 2017; Luthra et al., 2018; Chen et al., 2019; Kottkamp et al., 2019; Mei-jiao et al., 2019; Yang et al., 2019). However, most of these compounds lack verified drug targets making subsequent drug optimization and further application impossible (Zeng et al., 2005; Hoffmann et al., 2011; Lucas-Hourani et al., 2013; Raveh et al., 2013; Chung et al., 2016; Grandin et al., 2016; Lucas-Hourani et al., 2017; Luthra et al., 2018; Kottkamp et al., 2019). There are only a few inhibitors against pyrimidine synthesis that can be carried forward to animal studies, however, their antiviral efficacies were unsatisfactory or even ineffective at all (Zeng et al., 2005; Qing et al., 2010; Marschall et al., 2013; Raveh et al., 2013; Grandin et al., 2016; Cheung et al., 2017; Mei-jiao et al., 2019). For example, a pyrimidine synthesis inhibitor FA-613 without a specific target protected only 30.7% of mice from lethal influenza A virus infection when compared to the DAA drug Zanamivir (100%) in parallel (Cheung et al., 2017). Another two compounds, Cmp1 (Marschall et al., 2013) and FK778 (Zeng et al., 2005), which target DHODH, a rate-limiting enzyme in the fourth step of the *de novo* pyrimidine synthesis pathway, could only inhibit the DNA virus (CMV) replication in RAG^−/−^ mice, but their therapeutic effects on the upcoming diseases were unexplored. Therefore, more potent pyrimidine synthesis inhibitors, especially ones with the specific drug target, are urgent to be developed to prove whether such an HTA drug is valuable towards clinical use or has any advantages over DAA drugs in antiviral treatment.

**Figure 1:**
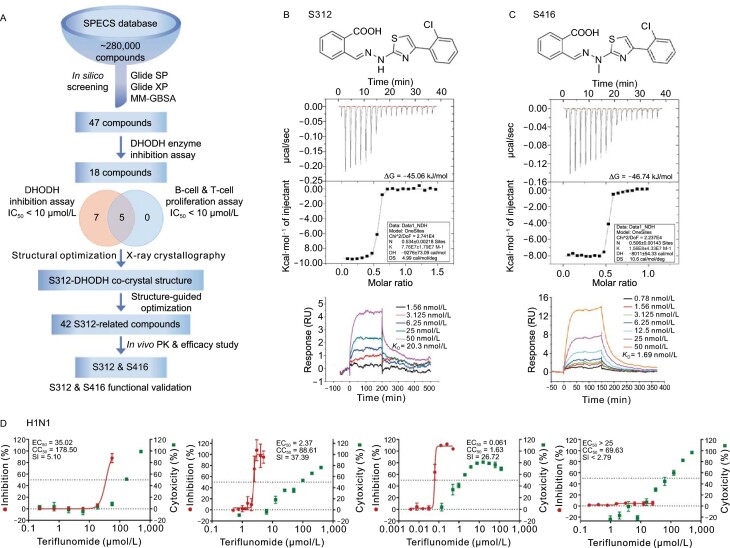

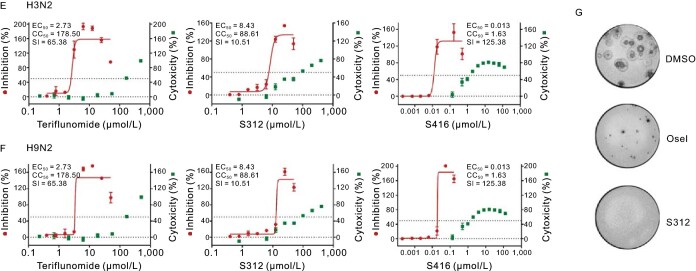
**Discovery of novel and potent DHODHi and their anti-influenza A virus activities**. (A) The discovery and design of S312 and S416. The detailed descriptions of the discovery workflow are in Method. Binding analysis of S312 (B) and S416 (C). Thermodynamic analysis of the binding of S312 and S416 to DHODH was carried out at 25 °C on a MicroCal iTC200 instrument. Kinetic analysis of the binding of S312 and S416 to DHODH was performed with a Biacore T200 instrument. (D) Inhibitory activities of DHODHi against influenza A virus (A/WSN/33 [H1N1]). MDCK cells were infected with WSN virus (20 TCID_50_/well) in the presence of increasing concentrations of DHODHi (Teriflunomide, Leflunomide, S312, and S416) for 72 h. Inhibition potencies (EC_50_) of these four compounds against the WSN virus and their cytoxicities (CC_50_) were all determined using cell viability assay. (E and F) Antiviral activities of DHODHi against influenza A virus H3N2 and H9N2. The experimental procedure and the detection method were the same as shown in (D). The results (D–F) are presented as a mean of at least three replicates ± SD. (G) Observation of virus morphology in the presence of the indicated compound. MDCK cells were treated with 5EC_50_ of S312 or Oseltamivir (EC_50_ = 0.64 μmol/L) in the meantime of WSN infection. Cells were fixed and stained after 48 h.p.i., Upper well, Control (DMSO); Centre well, Oseltamivir (3 μmol/L, ~5EC_50_); Bottom well, S312 (12.5 μmol/L, ~5EC_50_)

To identify potent and low-toxicity DHODH inhibitors (DHODHi), we previously conducted a hierarchal structure-based virtual screening (Fig. 1A) against ~280,000 compounds library towards the ubiquinone-binding site of DHODH (Diao et al., 2012). We finally obtained two highly potent DHODHi S312 and S416 with IC_50_ of 29.2 nmol/L and 7.5 nmol/L through structural optimization (Li et al., 2015; Zhu et al., 2015a), which are >10-folds potent than the FDA approved DHODHi Teriflunomide (IC_50_ of 307.1 nmol/L). By using these two potent inhibitors, we could fully evaluate DHODH as a valuable host target both in infected cells and *in vivo* in infected animals. We identified that targeting DHODH offers broad-spectrum antiviral efficacies against various RNA viruses, including the DAA-resistant influenza virus and the newly-emerged coronavirus SARS-CoV-2. Especially, our potent DHODHi can protect 100% mice from lethal influenza challenge, which is as good as the DAA drugs, and is even effective in the late phase of infection when DAA drug is no longer responding.

## Results

### The discovery of potent and selective DHODHi with low toxicity *in vivo*

By virtual screening, we identified two molecules with the same novel scaffold, which is obviously different from those of Leflunomide/Teriflunomide or Brequinar. The X-ray crystal structure of DHODH in complex with S416 (Fig. S1A; Table S1) further verified the binding mode of S416 at the ubiquinone-binding site of DHODH that is similar to S312. Compared to S312, the addition of the methyl group of S416 can perfectly occupy the small hydrophobic subsite formed by residues Met43, Leu46, and Gln47 on DHODH to form favorable Van der Waals interactions, contributing to the enhanced binding affinity of S416. The binding free energies for S312 and S416 were −45.06 kJ/mol and −46.74 kJ/mol, and the binding equilibrium dissociation constants (*K*_D_) were 20.3 and 1.69 nmol/L, respectively (Fig. 1B and 1C). Additionally, the two inhibitors exhibited a clear trait of fast-associating (*k*_on_) and slow-dissociating (*k*_off_) inhibition (Table S2), providing themselves as ideal drug candidates with a high level of target occupancy.

Considering the species selectivity, the lead compound, from which S312 and S416 were generated by the same scaffold of (*E*)-2-(2-benzylidenehydrazinyl)-4-phenylthiazole, is highly selective to *human* DHODH (IC_50_ = 0.11 μmol/L) over *Plasmodium falciparum* DHODH (IC_50_ > 10 μmol/L) (Diao et al., 2012) indicating that S312 and S416 have the same highly selective to *human* DHODH. Moreover, S312 and 416 displayed almost no kinase inhibitory activities at the concentration of 1 μmol/L (Fig. S1B and S1C) when profiled against a panel of >180 kinases, suggesting that S312 and S416 particular inhibited DHODH rather than other enzymes.

We further studied the pharmacokinetic profile of S312 and S416 *in vivo* (Table S3). The two inhibitors displayed a relatively wide distribution (Vd_ss_) of 0.35 L/kg (S312) and 0.14 L/kg (S416), which is closer to the body water volume (0.67 L/kg) than the blood volume (0.05 L/kg) (Davies and Morris, 1993), suggesting that they tend to distribute moderately to the tissue components. Besides, both S312 and S416 have a proper oral bioavailability (*F* = 22.75% and 76.28%, respectively).

As S312 and S416 showed proper half-lives (8.20 h and 9.12 h, respectively, Table S3), they may have less possibility to bring side effects from drug accumulation in the body. In the single-dose acute oral toxicity studies, S312 was well tolerated at the highest dose of 1,000 mg/kg in ICR mice (Fig. S2A and S2B).

The data in all suggest that S312 and S416 are highly potent and selective DHODHi with an ideal pharmacokinetic profile and low toxicity.

### The high antiviral activities of DHODHi in influenza A virus-infected cells

To examine the antiviral activities of these DHODHi, we use the influenza A virus as a model virus. The 20 TCID_50_ of a labor stain of A/WSN/33 (H1N1, WSN) was applied to infect MDCK cells, and serial dilutions of drugs (DMSO as controls) were added at the same time when the cells were infected. Drug effects were evaluated by quantification of cell viability in both infected and non-infected cells, and the half-maximal effective concentration (EC_50_) and the half-cytotoxic concentration (CC_50_) of the indicated drug were obtained accordingly. The selectivity index (SI) was calculated by CC_50_/EC_50_. As shown in Fig. 1D, the antiviral effect of Leflunomide is hardly detectable at the cell culture level (EC_50_ > 25 μmol/L). However, Teriflunomide, the active metabolite of Leflunomide, exhibited a clear antiviral effect against the WSN virus (EC_50_ = 35.02 μmol/L, CC_50_ = 178.50 μmol/L, SI = 5.10). As compared to Teriflunomide, the potent DHODHi S312 is ~15-fold stronger (EC_50_ = 2.37 μmol/L) and S416 is ~574-fold stronger (EC_50_ = 0.061 μmol/L) than Teriflunomide in their antiviral activities. We also tested different influenza A virus subtypes of H3N2 and H9N2 (summarized in Table 1). The antiviral effective curves of Teriflunomide, S312, and S416 to H3N2 and H9N2 were shown also in MDCK cells (Fig. 1E and 1F). The antiviral activity of S416 was again the highest, followed by Teriflunomide and S312. When we compared the drug effects by virus plaque assay, the results in Fig. 1G showed that the positive control DAA drug Oseltamivir (Osel) could reduce the plaque size to needlepoint size. However, the virus plaque in equivalent S312-treatment was not observable at all, indicating that S312 is more efficient in inhibiting virus replication than Osel.

**Table 1:** Broad-spectrum antiviral potency of DHODHi

Compounds	EC_50_ (µmol/L)/CC_50_ (µmol/L)/SI	Viruses						
		A/WSN/33 (H1N1)	A/DongHu/06 (H3N2)	A/GuangZhou/99 (H9N2)	Zika	Ebola	SARS-CoV-2 (MOI = 0.03)	SARS-CoV-2 (MOI = 0.05)
S312	EC_50_	2.37 ± 0.08	8.43 ± 0.79	13.17 ± 3.09	1.24 ± 0.39	11.39 ± 2.41	1.59 ± 0.01	1.56 ± 0.32
	CC_50_	88.61 ± 9.40	88.61 ± 9.40	88.61 ± 9.40	62.07 ± 20.99	118.70 ± 34.70	158.20 ± 20.67	158.20 ± 20.67
	SI	37.39	10.51	6.73	50.06	10.42	99.50	101.41
S416	EC_50_	0.061 ± 0.136	0.013 ± 0.006	0.021 ± 0.001	0.019 ± 0.003	0.018 ± 0.010	0.014 ± 0.0001	0.017 ± 0.002
	CC_50_	1.63 ± 0.35	1.63 ± 0.35	1.63 ± 0.35	54.73 ± 23.56	85.43 ± 2.00	178.60 ± 16.38	178.60 ± 16.38
	SI	26.72	125.38	77.62	2880.53	4746.11	12757.14	10505.88
Brequinar	EC_50_	0.241 ± 0.019	0.022 ± 0.001	0.060 ± 0.002	0.304 ± 0.010	0.102 ± 0.011	0.060 ± 0.012	0.123 ± 0.003
	CC_50_	2.87 ± 0.18	2.87 ± 0.18	2.87 ± 0.18	52.70 ± 21.18	13.45 ± 1.30	231.30 ± 20.92	231.30 ± 20.92
	SI	11.91	130.45	47.83	173.36	131.86	3855.00	1880.49
Teriflunomide	EC_50_	35.02 ± 1.25	2.73 ± 0.32	3.36 ± 0.11	17.72 ± 1.57	3.41 ± 0.95	6.00 ± 0.77	26.06 ± 6.40
	CC_50_	178.50 ± 18.07	178.50 ± 18.07	178.50 ± 18.07	55.68 ± 8.70	110.00 ± 22.52	850.50 ± 67.69	850.50 ± 67.69
	SI	5.10	65.38	53.13	3.14	32.26	141.75	32.64
Leflunomide	EC_50_	>25.00	—	—	—	—	—	41.49 ± 8.84
	CC_50_	69.63 ± 17.85	—	—	—	—	—	879.00 ± 62.58
	SI	<2.79	—	—	—	—	—	21.19

SI value was equal to CC_50_/EC_50_; “—” indicated not tested

These results indicate that DHODHi, especially S312 and S416 exhibited direct antiviral activities to different subtypes of influenza A viruses by shutting off virus replication more efficiently than Osel.

### The broad-spectrum antiviral activities of DHODHi to Zika, Ebola and newly-emerged SARS-CoV-2 viruses in infected cells

As all infectious viruses rely on actuating cellular pyrimidine synthesis process to replicate, it is reasonable to speculate that DHODHi have broad-spectrum antiviral efficacies. We, therefore, tested several highly impacted acute infectious RNA viruses. All compounds of Teriflunomide, Brequinar, S312, and S416 showed inhibitory effects against the Ebola virus (EBOV) mini-replicon, with EC_50_ of 3.41, 0.102, 11.39 and 0.018 μmol/L, respectively (Fig. S3A). To our surprise, S416 showing relatively high cytotoxicity in MDCK cells (CC_50_ = 1.63 μmol/L in Fig. 1D) was less toxic to EBOV-mini-replicon supporting BSR-T7/5 cells (CC_50_ = 85.43 μmol/L). Thus, a significantly high SI = 4 746.11 was achieved by S416. We subsequently tested the inhibitory effects of DHODHi against the Zika virus (Fig. S3B). EC_50_ values were 17.72, 0.304, 1.24 and 0.019 μmol/L for Teriflunomide, Brequinar, S312 and S416, respectively. Again, the selective index of S416 was the highest of SI = 2 880.53.

**Figure 2:**
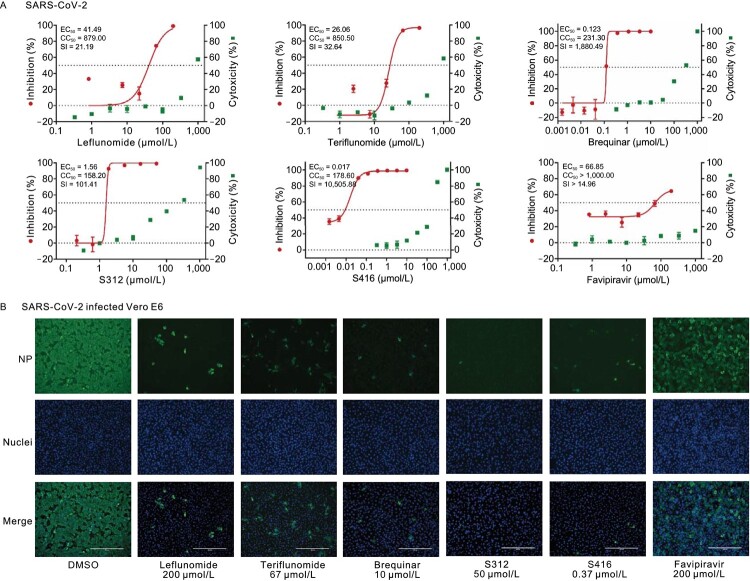
**Antiviral activities of DHODHi against SARS-CoV-2** ***in vitro***. (A) Anti-SARS-CoV-2 virus potency. Aliquots of Vero E6 cells were seeded in 96-well plates and then infected with Beta CoV/Wuhan/WIV04/2019 at MOI of 0.05. At the same time, different concentrations of the compounds were added for co-culture. Cell supernatants were harvested 48 h.p.i. and RNA was extracted and quantified by qRT-PCR to determine the numbers of viral RNA copies. Inhibition potencies (EC_50_) of the compounds were determined by percentage viral RNA reductions as compared to control treatment (DMSO), and their cytoxicities (CC_50_) were determined using cell viability assay. The results are presented as a mean of at least three replicates ± SD. (B) Immuno-fluorescence assay of SARS-CoV-2-infected cells. Vero E6 cells were infected with SARS-CoV-2 under the same procedure of (A). Cells were fixed and permeabilized for staining with an anti-viral NP antibody, followed by staining with Alexa 488-labeled secondary antibody. Green represents infected cells. Nuclei were stained by DAPI, and the merge of NP and nuclei were shown. Scale bar, 400 μm

During the preparation of this manuscript, a severe outbreak of SARS-CoV-2 had occurred in December 2019, and we responded quickly to examine the antiviral activities of DHODHi against this new coronavirus. The data in Fig. 2A showed that Teriflunomide had a solid antiviral potency of EC_50_ = 26.06 μmol/L (at MOI = 0.05), ~2.6-fold stronger than Favipiravir (EC_50_ = 66.85 μmol/L), whereas its pro-drug Leflunomide showed less inhibition of EC_50_ = 41.49 μmol/L. Additionally, Brequinar, S312, S416 and exhibited ideal antiviral potencies of EC_50_ = 0.123 μmol/L (SI = 1 880.49), EC_50_ = 1.56 μmol/L (SI = 101.41), and EC_50_ = 0.017 μmol/L (extensively high SI = 10 505.88) at MOI = 0.05, respectively (Fig. 2A). Compared with our previous publication of Remdesivir (EC_50_ = 0.77 μmol/L, SI > 129.87) and Chloroquine (EC_50_ = 1.13 μmol/L, SI > 88.50) (Wang et al., 2020), which are currently used in clinical trials against SARS-CoV-2, S416 had much greater EC_50_ and SI values (66.5-fold stronger than Chloroquine in EC_50_) against SARS-CoV-2. To determine more carefully the infection-dose dependent antiviral activities of DHODHi, a bit low MOI of 0.03 (Fig. S3C) was applied. Teriflunomide, which can be transferred to clinical treatment of SARS-CoV-2 immediately as an approved drug, showed lower EC_50_ of 6.00 μmol/L and SI = 141.75 at MOI of 0.03, indicating that Teriflunomide with effective EC_50_ and SI values have all the potentials to treat SARS-CoV-2-induced COVID-19 disease as an “old drug in new use” option. The antiviral activities of Brequinar, S312, and S416 were similar at the two MOIs indicating that they are enough potent to take effects at low concentrations.

We further did immuno-florescent assay to visualize the drug potencies against SARS-CoV-2. The data in Fig. 2B clearly showed that all DHODHi including Leflunomide (200 μmol/L), Teriflunomide (67 μmol/L), Brequinar (10 μmol/L), S312 (50 μmol/L), S416 (0.37 μmol/L) exhibited similar inhibitions on SARS-CoV-2, corresponding to the strength of their EC_50_ values. By contrast, even 200 μmol/L of Favipiravir only inhibited partial SARS-CoV-2 infections, indicating that DHODHi are more efficient inhibitors over Favipiravir to SARS-CoV-2 in this assay. Again, S416 turns to be the best efficient chemical so far against SARS-CoV-2 at the cellular level.

### S312 exhibited equivalent antiviral efficacy to Oseltamivir in influenza A virus-infected animals

**Figure 3:**
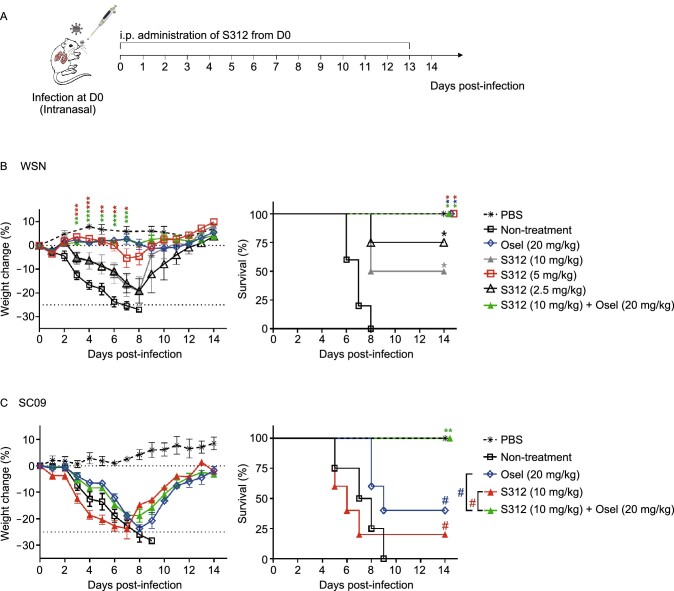
**The** ***in vivo*** **antiviral activity of S312 in influenza A virus-infected mice**. (A) Diagram of the experimental procedure. (B) BALB/c mice were intranasal infected with 4,000 PFU of WSN virus and intraperitoneal injected (i.p.) with PBS, S312 (2.5, 5, 10 mg/kg), Oseltamivir (20 mg/kg) and S312 + Oseltamivir (10 mg/kg + 20 mg/kg) around 3 h before infection. The compounds were continuous given once per day from D0-D13 respectively. The body weight and survival were monitored for 14 days or until body weights lost more than 25% (non-treatment group *n* = 5, the other groups *n* = 4). (C) Same procedure as (A). BALB/c Mice were inoculated intranasally with 300 PFU of A/SC/09 (H1N1) and i.p. with PBS, S312 (10 mg/kg), Oseltamivir (20 mg/kg) and S312 + Oseltamivir (10 mg/kg + 20 mg/kg) once per day from D0 to D13. The body weight and survival were monitored until 14 days post-infection or when the body weights lost more than 25% (PBS group *n* = 3, non-treatment group *n* = 4, the other groups *n* = 5). The dotted lines indicated initial weight and endpoint for mortality (25% weight loss) separately. The body weights are present as the mean percentage of weight change ±SEM and survival curves are shown. Asterisks (*) indicate significance when the indicated group compared with the non-treatment group, and pound signs (#) indicate significance between single treatment and combined treatment. *#*P* < 0.05; **P < 0.01; and ***P < 0.001. Statistical analysis, two-way ANOVA for weight curves, and the log-rank test for survival curves

In all the previous studies, inhibitors to DHODH or pyrimidine synthesis pathway were only active *in vivo* at low efficacies showing no obvious superior to DAA drugs. To test whether our potent DHODHi could refresh the important role of targeting DHODH in viral diseases, we next explored *in vivo* efficacy of S312 by intranasal infecting BALB/c mice at a lethal dose of WSN (2 LD_50_ = 4,000 PFU) or 2009 pandemic H1N1 (A/Sichuan/01/2009, SC09) (2 LD_50_ = 300 PFU) virus. Treatment of S312 or Osel or combined treatment of “S312 + Osel” was given by the intraperitoneal (i.p.) route around 3 h before infection once per day from D0 to Day 13 to determine body weight change and mortality (experimental procedure shown in Fig. 3A). The data in Fig. 3B showed that the body weights of mice from the non-treatment infected group all dropped more than 25% and died at D8 p.i. DAA drug of Osel could indeed totally rescue all the mice from body weight loss and death. Equivalently, S312 (5 mg/kg, red line) was also able to confer 100% protection and little body weight loss similar to Osel. S312 at 2.5 mg/kg and 10 mg/kg could confer 75% protection and 50% protection, respectively. The results suggest that S312 of a modest dose (5 mg/kg) would achieve the equivalent 100% protection to DAA drug when used from the beginning of the infection. According to the pharmacokinetic data of 10 mg/kg S312 (C_max_ = 5 310.50 μg/L = ~14.84 μmol/L) (Table S3) which is sufficient to afford all of the effective concentrations in Table 1 (especially EC_50_ = 2.37 μmol/L against the WSN), we mainly used 10 mg/kg and the modest dose of 5 mg/kg in the following experiments.

To further prove S312 is also effective to treat a natural-isolated H1N1 virus, the 2009 pandemic H1N1 strain, SC09, was used to infect mice. The data in Fig. 3C showed that even though SC09 is less sensitive to either Osel (40% protection) or S312 (20% protection) compared to WSN, 100% protection could still be achieved in combined treatment of S312 + Osel, indicating that HTA and DAA drug combination can augment therapeutic effects.

Except for broad activity to different viruses, HTA drug such as DHODHi has another advantage over DAA drug to overcome drug-resistant (Table 2). To prove this, we generated a current-circulating Oseltamivir-resistant NA^H275Y^ mutant virus (in WSN backbone) by reverse genetics (Fig. S4A and S4B). We found that the NA^H275Y^ virus did not respond to Osel (20 mg/kg/day)-treatment at all, but 2.5 mg/kg/day of S312 can rescue 50% of mice from lethal infection of NA^H275Y^ virus (Fig. S4C).

**Table 2:** Antiviral potency of DHODHi against Osel-resistant strain

Virus type	EC_50_ (µmol/L)	
	S312	Oseltamivir
NAmut^H275Y^	5.967	~1,046
Wild type	3.077	43.01

The data above refresh DHODH as an attractive host target in treating viral diseases with equivalent efficacy to DAA drug and is more advantageous when facing DAA-drug-resistant viruses.

### DHODHi inhibit virus replication through interrupting the *de novo* pyrimidine synthesis pathway

**Figure 4:**
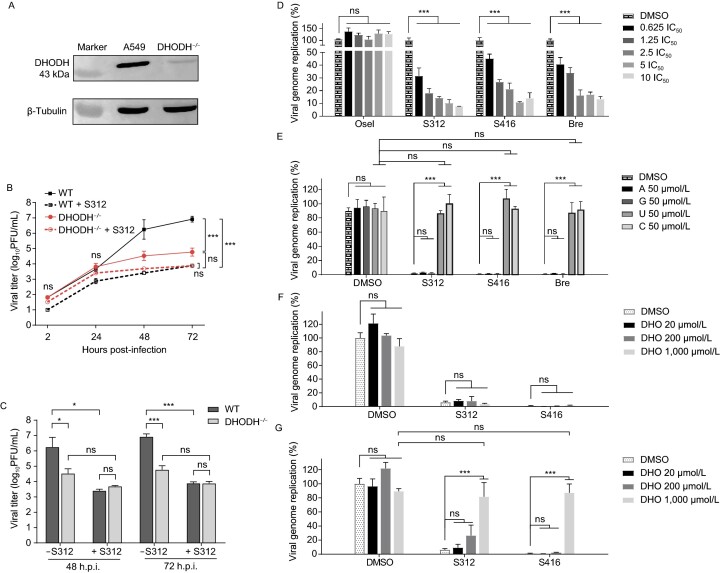
**DHODHi inhibit virus replication through interrupting the** ***de novo*** **pyrimidine synthesis pathway**. (A) The expression levels of DHODH protein in WT and DHODH^−/−^ A549 cells. (B) The growth curve of the WSN virus with or without treatment of S312 on A549 (WT and DHODH^−/−^) cells. The A549 cells (WT and DHODH^−/−^) were infected with WSN virus (MOI of 0.01) and treated with S312 at a final concentration of 12.5 μmol/L (~5EC_50_). The supernatants were assayed for viral titers at 2 h, 24 h, 48 h, and 72 h post-infection by plaque assay. (C) The statistic analysis for (B). (D) The 293FT cells were co-transfected with the influenza virus minigenome plasmid system (PB1, PB2, PA, NP, pPoII-NP-luc, and pRLSV40). After 12 h.p.i., cells were treated with 2-fold serial dilutions of Oseltamivir, S312, S416, and Brequinar respectively. The luciferase activities were measured 24 h of post-treatment. (E) Effects of nucleosides addition on the antiviral efficacies of S312, S416, and Brequinar. Around 10-fold EC_50_ of S312 (24 μmol/L) or S416 (0.6 μmol/L) or Brequinar (2.5 μmol/L) and 50 μmol/L four nucleosides (Adenosine, Uridine, Cytidine, Guanosine) were added at the same time on 293FT cells. The luciferase activities were measured 24 h post-treatment. (F and G) Effects of addition of dihydroorotate (DHO) or Orotic acid (ORO) on the antiviral efficacies of S312 and S416. The luciferase activities were detected as above after treating with indicated concentrations of DHO or ORO. All results are presented as a mean of three replicates ± or + SD. Statistical analysis, two-way ANOVA for (B) and (C). One-way ANOVA for (D–G). ns, *P* > 0.05; *P < 0.05; ***P < 0.001

To elucidate the essential role of DHODH in the viral replication cycle, we generated a DHODH^−/−^ A549 cell line by CRISPR-Cas9 gene knock-out (KO) technology (Figs. S5A and 4A). Unexpectedly, the cell proliferation rate was barely affected in DHODH^−/−^ cells indicating DHODH is not indispensable for cell growth at least for three days (72 h) (Fig. S5B). By contrast, as compared to wild-type (WT) A549 cells, virus growth was largely inhibited in DHODH^−/−^ cells with a 132-fold reduction of infectious particles at 72 h post-infection (h.p.i.) (Fig. 4B, black solid line vs. red solid line). When S312 (5EC_50_) was added into the culture medium, dramatic reduction of virus growth only occurred in WT cells but not in DHODH^−/−^ cells (Fig. 4B, differs between black lines vs. differs between red lines). Moreover, in the presence of S312, there were no differences in virus titers between WT and DHODH^−/−^ cells (Fig. 4B, blank dotted line vs. red dotted line) indicating that S312 did not act in the absence of DHODH. The statistical analysis of Fig. 4B was also shown in Fig. 4C in detail. These results together prove that virus growth but not the coincident cell growth requires DHODH activity, and antiviral action of S312 is implemented by particularly targeting DHODH.

The general virus growth cycle includes virus entry, viral genome replication, and virus release. To further validate whether virus genome replication is the major target of DHODHi, we used the influenza A virus mini-replicon system to quantify viral genome replication. Brequinar, another potent inhibitor of DHODH was included as a positive control (Chen et al., 1992), whereas Osel targeting the influenza NA protein served as a negative control. The results in Fig. 4D showed no inhibition on viral genome replication in the Osel-treated group, but there were obvious inhibitions on viral genome replication in both S312- and S416-treated groups as well as Brequinar-treated group in dose-dependent manners. Almost 90% of viral genome replication was suppressed by 10EC_50_ of S312 (24 μmol/L) and S416 (0.6 μmol/L). As DHODH catalyzes the oxidation of dihydroorotate (DHO) to produce orotic acid (ORO) and finally forms UTP and CTP, we added four nucleosides (Adenosine (A), Guanosine (G), Uridine (U), and Cytidine (C)), DHO, ORO respectively to the mini-replicon system to identify the target of S312 and S416. The results in Fig. 4E showed that the addition of 50 μmol/L of either U or C could effectively rescue viral genome replication in S312- and S416-treated cells (as well as in Brequinar-treated cells), whereas addition of neither A nor G changed the inhibitory effects. Moreover, there were no differences in viral genome replication when U or C was complemented into S312- or S416-treated cells as compared to untreated cells (DMSO), indicating that these compounds only interrupted the pyrimidine *de novo* synthesis but not off-targeted to enzymes in the salvage pathways wherein extracellular pyrimidine nucleosides must serve as templates (Fig. 4E). A supplement of DHODH substrate DHO could not rescue viral genome replication (Fig. 4F), but a supplement of DHODH product ORO could gradually reverse the inhibition effects of S312 and S416 (Fig. 4G). Further statistical analysis showed no differences in viral genome replication when 1,000 μmol/L ORO was complemented into S312- and S416-treated cells as compared to untreated cells (DMSO), indicating that these compounds did not off-target to enzymes located downstream of ORO (Fig. 4G). The results in all further confirmed that compounds S312 and S416 inhibit viral genome replication via targeting DHODH and interrupting the fourth step in *de novo* pyrimidine synthesis.

### S312 is advantageous over the DAA drug to treat advanced and late-phase disease with decreasing cytokine/chemokine storm

**Figure 5:**
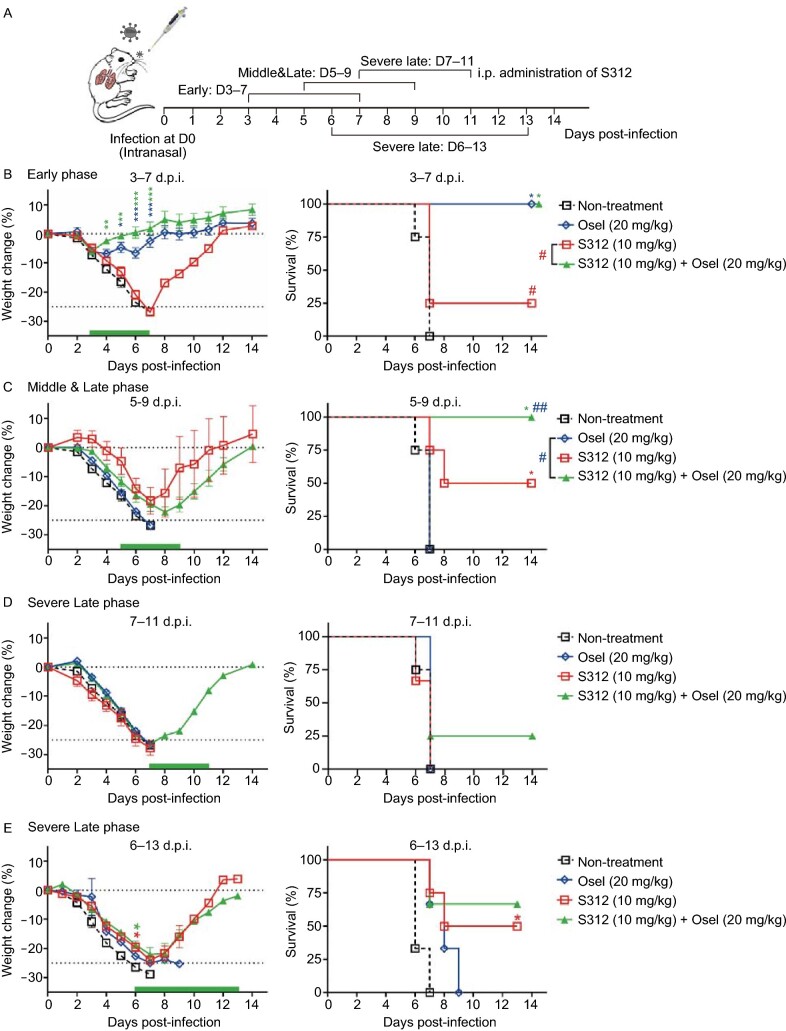
**S312 is more effective at the late and severe infection phase as compared to DAA drug Oseltamivir**. (A) Diagram of the experimental procedure. (B–D) BALB/c mice were inoculated intranasally with 4000 PFU of WSN virus and then i.p. with S312 (10 mg/kg), Oseltamivir (20 mg/kg), or S312 + Oseltamivir (10 mg/kg + 20 mg/kg) once per day from D3-D7 (B), D5-D9 (C), D7-D11 (D). Another groups of S312 (5 mg/kg), Oseltamivir (20 mg/kg) or S312 + Oseltamivir (5 mg/kg + 20 mg/kg) were given i.p. once per day from D6 to D13 in (E). The green bars indicate the period of drug administration. The body weight and survival were monitored until 14 days post-infection or when the body weight lost more than 25%. (B and C) *n* = 4 per group; (D) S312 (10 mg/kg) *n* = 3, the other groups *n* = 4; (E) S312 (5 mg/kg) *n* = 4, the other groups *n* = 3. The dotted lines indicate initial weight and endpoint for mortality (25% weight loss) separately. The body weights are present as the mean percentage of weight change ±SEM and survival curves were shown. Asterisks (*) indicate significance when the indicated group compared with the non-treatment group, and pound signs (#) indicate significance between single treatment and combined treatment. *#*P* < 0.05; **##*P* < 0.01; and ***P < 0.001. Statistical analysis, two-way ANOVA for weight curves, and the log-rank test for survival curves

It is documented elsewhere that DAA drugs such as Osel are only completely effective in the early phase of infection, optimally within 48 h of the onset of the symptom (McNicholl and McNicholl, 2001). Till now, there is no approved drug to treat advanced influenza disease at the late phase specifically. We supposed that S312 could be effective in the middle or late phase of disease because it targets a host pro-viral factor of DHODH not affected by the viral replication cycle. To test this, we compared the therapeutic windows of S312 and Osel in early (D3-D7), middle & late (D5-D9), and severe late (D7-D11 or D6-D13) phases (workflow is shown in Fig. 5A). When drugs were given in the early phase, both Osel-treatment and “Osel + S312”-combination-treatment conferred 100% protection (Fig. 5B). When drugs were given at the middle & late phase (Fig. 5C), single Osel-treatment wholly lost its antiviral effect with no surviving. However, S312-treatment could provide 50% protection, and drug combination reached to 100% protection. When drugs were given at severe late phase of the disease that mice were starting dying (Fig. 5D), neither single treatment of Osel nor S312 could rescue the mice from death, but combined treatment still conferred to 25% survival. To define the advantage of S312 in treating severe disease, we additionally treated the mice a bit early before dying at around 20% body weight loss (D6-D13) with a more optimal dose of S312 (5 mg/kg). The data in Fig. 5E showed that S312 rescued 50% of mice from severe body-weight losses, and combined treatment conferred an additional 16.7% survival. These results once again highlight that S312 has remarkable advantages over Osel to treat severe diseases at the late phase, and its therapeutic effectiveness could even be improved when S312 was combined with DAA drug.

It is known that severe acute infections, including influenza and COVID-19, always induce pathogenic immunity as cytokine/chemokine storms. Leflunomide and Teriflunomide are already clinically used in autoimmune disease to inhibit pathogenic cytokines and chemokines. We, therefore, suspected that DHODHi should also prevent cytokine-storm in viral infectious disease. BALF from either Osel- or “S312 + Osel”-treated mice were collected at D14 in an independently repeated severe late-phase treatment with a lower infection dose (1 LD50, 2000 PFU) to allow partial survival from Osel-treatment. Comparable body weights of the survival mice could exclude the differences in virus load (Fig. S6A). The data in Fig. S6B showed that the pathogenic inflammatory cytokines in “S312 + Osel”-treated group was largely reduced as compared to Osel-treated mouse in the levels of IL6, MCP-1, IL5, KC/GRO (CXCL1), IL2, IFN-γ, IP-10, IL9, TNF-α, GM-CSF, EPO, IL12p70, MIP3α and IL17A/F (listed in the order of reducing significance).

Furthermore, we tested the drug efficacy of S416 in Zika virus-infected AG6 adult mice. The data in Fig. S7A showed that S416 (10 mg/kg) could successfully rescue 25% of mice from high lethal dose infection of the Zika virus. Interestingly, the only survival mouse is male (Fig. S7B) but not female (Fig. S7C), which coincide with previous findings that female mice and women were more susceptible to Zika infection (Coelho et al., 2016; Tang et al., 2016; Carroll et al., 2017).

In summary, the above results provide striking information that DHODHi are effective in infected animals not only by inhibiting virus replication but also by eliminating excessive cytokine/chemokine storm, which suggests the usage of DHODHi could be beneficial to the advanced stage of disease at late-phase infection.

## Discussion

Many different RNA viruses had caused epidemics and pandemics in the past 20 years, from avian influenza virus (AIV) H5N1 in 1997, SARS-CoV in 2002, pandemic H1N1 in 2009, MERS-CoV in 2012, AIV H7N9 in 2013, Ebola virus in 2013, Zika virus in 2016, to SARS-CoV-2 in 2019. Many of them are still prevalent in certain countries and SARS-CoV-2 currently continues to cause huge devastation worldwide. The conventional one-bug-one-drug paradigm is insufficient to deal with the challenges of these emerging and re-emerging pathogenic viruses (Zhu et al., 2015b), moreover, it is too late to develop drugs against each virus behind epidemics (Debing et al., 2015). Thus, it is imperative to develop new ideas, highly effective, broad-spectrum antiviral agents, that can treat various viral infections. In this study, we applied DHODHi including a computer-aided designed compound S312 into viral infectious disease. We found that direct-targeting DHODHi are broad-spectrum antiviral both in cell culture and *in vivo*. The candidate S312 had further advantage to be used in infected animals with low toxicity and high potency. Moreover, S312 can rescue severe influenza infection by limiting inflammatory cytokine storm *in vivo*.

DHODH is a rate-limiting enzyme catalyzing the fourth step in pyrimidine *de novo* synthesis. It catalyzes the dehydrogenation of dihydroorotate (DHO) to orotic acid (ORO) to finally generate Uridine (U) and Cytosine (C) to supply nucleotide resources in a cell. Under normal conditions, nucleotides are supplied via both *de novo* biosynthesis and salvage pathways, the latter of which is a way of recycling pre-existing nucleosides from food or other nutrition. However, in virus-infected cells, a large intracellular nucleotide pool is demanded by rapid viral replication. It is therefore reasonable that *de novo* nucleotides biosynthesis rather than salvage pathway is more critical for virus replication. Our data indeed show that virus replication is largely restricted when the DHODH gene was knocked out even with a complete culture medium. By contrast, cell growth was not affected by lacking DHODH at all, indicating that *de novo* nucleotides biosynthesis is not indispensable in normal cell growth without infection at least for days. More interestingly, we notice that compared with DNA viruses, RNA viruses need unique UMP but not TMP in their genomes. UMP is the particular nucleoside produced by DHODH, which means RNA viruses might be more sensitive to DHODH activity. SARS-CoV-2, for instance, has around 32% of UMP in its genome explaining why DHODHi are effective and superior to SARS-CoV-2. Nevertheless, the comparison between different viruses is worth to be studied in the future.

Although several DHODHi have been documented to be antiviral by high-throughput screening (Harvey et al., 2009; Wang et al., 2011; Adcock et al., 2017; Cheung et al., 2017). Most of these compounds are investigated at cell culture level with unknown *in vivo* efficacies. Therefore, the development of broad-spectrum antiviral agents targeting DHODH is still an exciting avenue in antiviral research. S312 and S416 present more potent inhibition and favorable pharmacokinetic profiles, moreover, the half-lives of S312 and S416 (8.20 h and 9.12 h, respectively) are much shorter and more appropriate than that of Teriflunomide, indicating that they may have less possibility to bring toxic side effects from drug accumulation in the body. Strikingly, S312 showed active effects *in vivo* in lethal dose infection of influenza A viruses not only when used from the beginning of infection but also in the late phase when DAA drug is not responding anymore. Another surprise is the high SI value of S416 against Zika (SI = 2 880.53), Ebola (SI = 4 746.11), and the latest SARS-CoV-2 (SI > 12,757.14). These data interpreted that S416 is highly promising to develop further as it should be to S312. The extremely high SI of S416 may be due to its high binding affinity and favorable occupation of the ubiquinone-binding site of DHODH with faster-associating characteristics (*k*_on_ = 1.76 × 10^6^ mol^−1^·s^−1^) and slower dissociating binding characteristic (*k*_off_ = 2.97 × 10^−3^ s^−1^), which will reduce the possibility of off-target *in vivo*. Moreover, EC_50_ values of S416 against the tested viruses are also lower than S312 at the double-digit nanomolar range (13–61 nmol/L). Unlike S312, the C_max_ of S416 is >1,000-fold of its antiviral EC_50_s at the cellular level. Considering the complexity of the *in vivo* antiviral experiments, the similar chemical structure, and the same target of S312 and S416, S416 was not enrolled as much as S312 in this study but will be in our future work.

Acute viral infections usually cause severe complications associated with hyper induction of pro-inflammatory cytokines, which is also known as “cytokine storm” firstly named in severe influenza disease (Yokota, 2003; Yuen and Wong, 2005). Several studies showed that seriously ill SARS patients expressed high serum levels of pro-inflammatory cytokines (IFN-γ, IL-1, IL-6, IL-12, and TGFβ) and chemokines (CCL2, CXCL10, CXCL9, and IL-8) compared to uncomplicated SARS patients (Wong et al., 2004; Zhang et al., 2004; Wang et al., 2005; Chien et al., 2006). Similarly, in severe COVID-19 cases, ICU patients had higher plasma levels of IL-2, IL-7, IL-10, GSCF, MCP1, MIP1A, and TNFα compared to non-ICU patients (Huang et al., 2020). Moreover, A clinical study of 123 patients with COVID-19 showed that the percentage of patients with IL-6 above normal is higher in the severe group (Wan et al., 2020). In terms of treatment, immunomodulatory agents can reduce mortality and organ injury of severe influenza. However, these immunomodulatory are mostly non-specific to viral infection but rather a systemic regulation, such as corticosteroid, intravenous immunoglobulin (IVIG) or angiotensin receptor blockers (Luke et al., 2006; Liu et al., 2009; Gao et al., 2013; Hung et al., 2013). Leflunomide and its active metabolite Teriflunomide have been approved for clinical treatment for excessive inflammatory diseases such as rheumatoid arthritis and multiple sclerosis (Munier-Lehmann et al., 2013). Our data once again proved that DHODHi could further reduce cytokine storm over DAA drugs when using influenza A virus-infected animal as a model. We believe that a similar immune-regulating role of DHODHi will exist in COVID-19 patients.

The active metabolite A771726 (Teriflunomide) of Leflunomide inhibited the proliferation of PHA mitogen-stimulated human lymphocytes with the IC_50_ value of 46 µmol/L (Fox et al., 1999). The recommended dosage of Leflunomide in treating rheumatoid arthritis (RA) is loading 100 mg/day for 3 days, and subsequently administering 10 mg or 20 mg once daily. Thus, the blood concentration of this clinical dose is 66.62 ± 35.53 µmol/L (18 ± 9.6 mg/L) (Rozman, 2002), which could be sufficient to afford its therapeutic concentration of anti-RA (46 µmol/L), as well as anti-SARS-CoV-2 (Teriflunomide: EC_50_ = 26.06 μmol/L at MOI = 0.05; EC_50_ = 6.00 μmol/L at MOI = 0.03). As Leflunomide has been widely used and proved to treat RA with clinical safety and efficacy, it could be also feasible for the clinical trial for the treatment of COVID-19.

Thus, by targeting DHODH, the single key enzyme in viral genome replication and immune-regulation, a dual-action of DHODH can be realized in fighting against a broad spectrum of viruses and the corresponding pathogenic-inflammation in severe infections. We hope the results of our study may ultimately benefit the patients who are now suffering from severe COVID-19 and other infectious diseases caused by emerging and re-emerging viruses.

## Electronic supplementary material

The online version of this article (https://doi.org/10.1007/s13238-020-00768-w) contains supplementary material, which is available to authorized users.

## Supplementary Material

13238_2020_768_MOESM1_ESM

13238_2020_768_MOESM2_ESM

## Abbreviations

A, adenosine; AIV, avian influenza virus; ATCC, American Type Culture Collection; BSA, broad-spectrum antivirals; C, cytidine; CC_50_, the half-cytotoxic concentration; DAA, direct-acting antiviral; DHO, dihydroorotate; DHODH, dihydroorotate dehydrogenase; DHODHi, DHODH inhibitors; EBOV, Ebola virus; EC_50_, the half maximal effective concentration; G, guanosine; HTAs, host-targeting antivirals; IVIG, intravenous immunoglobulin; *K*_D_, the binding equilibrium dissociation constant; KO, knock-out; ORO, orotic acid; Osel, Oseltamivir; qRT-PCR, real-time quantitative reverse transcription PCR; RA, rheumatoid arthritis; SI, selectivity index; U, Uridine; WT, wild-type.
