# Correction to: Novel and potent inhibitors targeting DHODH are broad-spectrum antivirals against RNA viruses including newly-emerged coronavirus SARS-CoV-2

**DOI:** 10.1007/s13238-020-00792-w

**Published:** 2020-10-08

**Authors:** Rui Xiong, Leike Zhang, Shiliang Li, Yuan Sun, Minyi Ding, Yong Wang, Yongliang Zhao, Yan Wu, Weijuan Shang, Xiaming Jiang, Jiwei Shan, Zihao Shen, Yi Tong, Liuxin Xu, Yu Chen, Yingle Liu, Gang Zou, Dimitri Lavillete, Zhenjiang Zhao, Rui Wang, Lili Zhu, Gengfu Xiao, Ke Lan, Honglin Li, Ke Xu

**Affiliations:** 1 State Key Laboratory of Virology, College of Life Sciences, Wuhan University, 430072, Wuhan, China; 2 Shanghai Key Laboratory of New Drug Design, State Key Laboratory of Bioreactor Engineering, School of Pharmacy, East China University of Science and Technology, 200237, Shanghai, China; 3 State Key Laboratory of Virology, Wuhan Institute of Virology, Center for Biosafety Mega-Science, Chinese Academy of Sciences, 430071, Wuhan, China; 4 CAS Key Laboratory of Molecular Virology and Immunology, Institut Pasteur of Shanghai, University of Chinese Academy of Sciences, Chinese Academy of Sciences, 200031, Shanghai, China

Correction to: Protein Cellhttps://doi.org/10.1007/s13238-020-00768-w

In the original publication there are few errors in Figure 1. The correct Figure 1 is provided in this correction.

**Figure 1:**
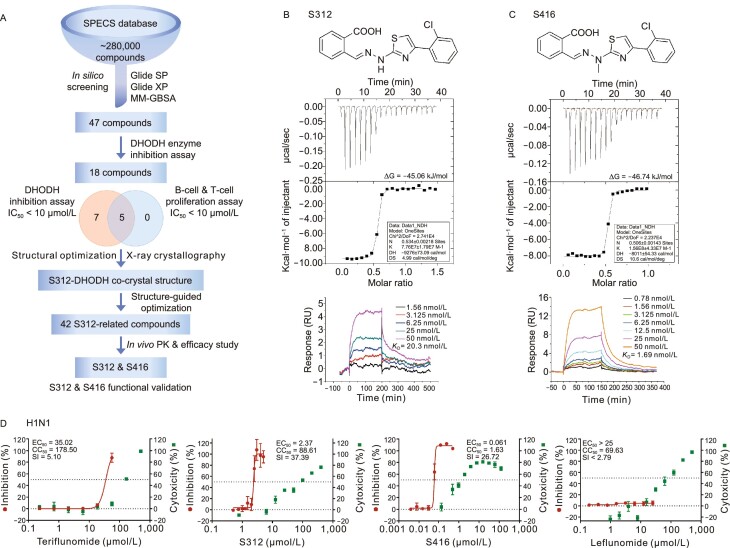

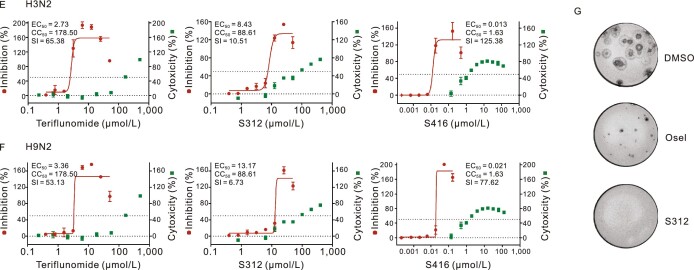
**Discovery of novel and potent DHODHi and their anti-influenza A virus activities**. (A) The discovery and design of S312 and S416. The detailed descriptions of the discovery workflow are in Method. Binding analysis of S312 (B) and S416 (C). Thermodynamic analysis of the binding of S312 and S416 to DHODH was carried out at 25 °C on a MicroCal iTC200 instrument. Kinetic analysis of the binding of S312 and S416 to DHODH was performed with a Biacore T200 instrument. (D) Inhibitory activities of DHODHi against influenza A virus (A/WSN/33 [H1N1]). MDCK cells were infected with WSN virus (20 TCID_50_/well) in the presence of increasing concentrations of DHODHi (Teriflunomide, Leflunomide, S312, and S416) for 72 h. Inhibition potencies (EC_50_) of these four compounds against the WSN virus and their cytoxicities (CC_50_) were all determined using cell viability assay. (E and F) Antiviral activities of DHODHi against influenza A virus H3N2 and H9N2. The experimental procedure and the detection method were the same as shown in (D). The results (D–F) are presented as a mean of at least three replicates ± SD. (G) Observation of virus morphology in the presence of the indicated compound. MDCK cells were treated with 5EC_50_ of S312 or Oseltamivir (EC_50_ = 0.64 μmol/L) in the meantime of WSN infection. Cells were fixed and stained after 48 h.p.i., Upper well, Control (DMSO); Centre well, Oseltamivir (3 μmol/L, ~5EC_50_); Bottom well, S312 (12.5 μmol/L, ~5EC_50_)

