# Correction to: Novel and potent inhibitors targeting DHODH are broad-spectrum antivirals against RNA viruses including newly-emerged coronavirus SARS-CoV-2

**DOI:** 10.1007/s13238-020-00801-y

**Published:** 2020-11-09

**Authors:** Rui Xiong, Leike Zhang, Shiliang Li, Yuan Sun, Minyi Ding, Yong Wang, Yongliang Zhao, Yan Wu, Weijuan Shang, Xiaming Jiang, Jiwei Shan, Zihao Shen, Yi Tong, Liuxin Xu, Yu Chen, Yingle Liu, Gang Zou, Dimitri Lavillette, Zhenjiang Zhao, Rui Wang, Lili Zhu, Gengfu Xiao, Ke Lan, Honglin Li, Ke Xu

**Affiliations:** 1 State Key Laboratory of Virology, College of Life Sciences, Wuhan University, Wuhan, 430072 China; 2 Shanghai Key Laboratory of New Drug Design, State Key Laboratory of Bioreactor Engineering, School of Pharmacy, East China University of Science and Technology, Shanghai, 200237 China; 3 State Key Laboratory of Virology, Wuhan Institute of Virology, Center for Biosafety Mega-Science, Chinese Academy of Sciences, Wuhan, 430071 China; 4 CAS Key Laboratory of Molecular Virology and Immunology, Institut Pasteur of Shanghai, University of Chinese Academy of Sciences, Chinese Academy of Sciences, Shanghai, 200031 China

## Correction to: Protein Cell 2020,11(10):723–739 10.1007/s13238-020-00768-w

In the original publication the author’s name ‘Dimitri Lavillete’ is published incorrectly. The correct author name should be spelt as ‘Dimitri Lavillette’ is provided in this correction.

