# Supplementary material for: Novel and potent inhibitors targeting DHODH are broad-spectrum antivirals against RNA viruses including newly-emerged coronavirus SARS-CoV-2
Source: Protein Cell. 2020 Aug 4;11(10):723–39. doi: 10.1007/s13238-020-00768-w (PMC7402641; doi:10.1007/s13238-020-00768-w)
Supplement: 13238_2020_768_MOESM2_ESM [file 13238_2020_768_moesm2_esm.pdf]

STAR★METHODS

Detailed methods are provided in the online version of this paper and include the following:

- KEY RESOURCES TABLE
- LEAD CONTACT AND MATERIALS AVAILABILITY
- EXPERIMENTAL MODEL AND SUBJECT DETAILS
  - ✧ Mice and animal housing
  - ✧ Influenza A virus infection model
  - ✧ Zika virus infection model
- METHOD DETAILS
  - ✧ Discovery of DHODHi
  - ✧ Isothermal titration calorimetry (ITC)
  - ✧ Surface plasmon resonance (SPR)
  - ✧ Crystallization and structure determination
  - ✧ Kinase-inhibition assays
  - ✧ *In vivo* drug toxicity study
  - ✧ Cell cultures, viral strains, and drugs
  - ✧ Anti-influenza virus activity
  - ✧ Detection of anti-ZIKV potency by real-time quantitative PCR
  - ✧ Bright-Glo Pharmacodynamic assay for Ebola Replicon
  - ✧ Detection of anti-SARS-CoV-2 potency by real-time quantitative PCR
  - ✧ IFA assay of SARS-CoV-2
  - ✧ Virus growth curve and plaque assay
  - ✧ Influenza mini-replicon system and DHODH substrates assay
  - ✧ Cytokine and chemokine measurements
- QUANTIFICATION AND STATISTICAL ANALYSIS
- DATA AND CODE AVAILABILITY

SUPPLEMENTAL INFORMATION

Supplemental Information includes four tables, seven figures, and one data file and can be found with this article online.

- Data S1. Results of the kinase inhibition assays for S312 and S416. Related to Fig. 1.

STAR★METHODS

KEY RESOURCES TABLE

REAGENT or RESOURCE	SOURCE	IDENTIFIER
Antibodies		
Mouse monoclonal anti-DHODH	Santa Cruz Biotechnology	Cat#sc-166348; RRID:AB_2091729
Rabbit polyclonal antibody anti bat SARS-related CoV NP	Wuhan Institute of Virology, CAS	N/A
Rabbit polyclonal anti-Influenza A virus NP conjugated with HRP	Antibody Research Center, Shanghai Institute of Biological Science	N/A
Bacterial and Virus Strains		
A/WSN/33(H1N1)	Han et al., 2014	N/A
A/WSN/33(H1N1)(NAmut^{H275Y})	This paper	N/A
A/Sichuan/1/2009(H1N1)	Laboratory of Yuelong Shu	N/A
A/Donghu/312/2006(H3N2)	Laboratory of Yuelong Shu	N/A
A/Guangzhou/333/99(H9N2)	Laboratory of Yuelong Shu	N/A
Zika virus SZ-WIV01, GENBANK: KU963796	Laboratory of Bo Zhang	N/A
2019BetaCoV/Wuhan/WIV04/2019, GENBANK: MN996528.1	Wuhan Institute of Virology, CAS	N/A
Chemicals, Peptides, and Recombinant Proteins		
S312	Li et al., 2015	N/A
S416	Song et al., 2016	N/A
Leflunomide	Sigma-Aldrich	L5025; CAS:75706-12-6
Teriflunomide	Sigma-Aldrich	SML0936; CAS: 163451-81-8
Brequinar	Sigma-Aldrich	B5707; CAS:96187-53-0
Oseltamivir	Sigma-Aldrich	BP903; CAS: 204255-11-8
Favipiravir	Selleck	S7975; CAS No.259793-96-9
Adenosine	Sigma-Aldrich	A4036; CAS:58-61-7
Uridine	Sigma-Aldrich	U3003; CAS: 58-96-8
Cytidine	Sigma-Aldrich	C4654; CAS: 65-46-3
Guanosine	Sigma-Aldrich	G6264; CAS:118-00-3
Orotic acid	Sigma-Aldrich	O2750; CAS: 65-86-1
Dihydroorotate	Sigma-Aldrich	D7128; CAS: 5988-19-2
Critical Commercial Assays		

Dual-Luciferase Assay Kit	Promega	Cat#E1910
Bright-Glo^{TM} Luciferase Assay	Promega	Cat#E2620
CellTiter-Glo® Cell Viability Assay	Promega	Cat#G7570
Cell Counting Kit-8	Beyotime	Cat#C0038
PrimeScript RT reagent kit	Takara	Cat#RR820A
U-PLEX Biomarker Group 1 (ms) 35-plex	Univ-Biotechnology	Cat#K15083K
Amine Coupling Kit	GE Healthcare	Cat#BR100050
Protein Thermal Shift Dye Kit	Thermo Fisher Scientific	Cat#4461146
Deposited Data		
DHODH-S416 complex structure	This paper	PDB: 6M2B
Experimental Models: Cell Lines		
Human: A549	ATCC	Cat#CCL-185; RRID:CVCL_0023
Human: Huh7	Laboratory of Dimitri Lavillette	N/A
Human: 293FT	Laboratory of Dimitri Lavillette	N/A
Cercopithecus aethiops: Vero E6	ATCC	Cat#CRL-1586; RRID:CVCL_0574
Canis familiaris: MDCK	ATCC	Cat#CCL-34; RRID:CVCL_0422
Hamster: BSR T7/5	Laboratory of Gang Zou	N/A
Human: A549 DHODH$^{-/-}$	This paper	N/A
Experimental Models: Organisms/Strains		
Mouse: BALB/c (Female)	Shanghai Lingchang biotechnology Co. Ltd	N/A
Mouse: ICR (Male)	Shanghai Sippr-BK laboratory animal Co. Ltd	N/A
Rat: Sprague-Dawley (Female; Male)	Shanghai Sippr-BK laboratory animal Co. Ltd	N/A
Mouse: AG6 (Female; Male)	Laboratory of Qibin Leng	N/A
Oligonucleotides		
Primers of SARS-CoV-2, see Table S4	This paper	N/A
Primers of Zika virus, see Table S4	This paper	N/A
Primers of DHODH sgRNAs, see Table S4	This paper	N/A
Primers of A/WSN/33(H1N1)(NAmut^{H275Y})	This paper	N/A
Recombinant DNA		
Plasmid: lenti-CRISPR v2	Laboratory of Dimitri Lavillette	N/A
Plasmid: psPAX2	Laboratory of Dimitri Lavillette	N/A
Plasmid: pMD2.G	Laboratory of Dimitri Lavillette	N/A

Plasmid: p3E5E-Luc	Jasenosky et al., 2010	N/A
Plasmid: pCEZ-NP	Jasenosky et al., 2010	N/A
Plasmid: pCEZ-VP35	Jasenosky et al., 2010	N/A
Plasmid: pCEZ-VP30	Jasenosky et al., 2010	N/A
Plasmid: pCEZ-L	Jasenosky et al., 2010	N/A
Plasmid: pPoⅡ-NP-luc	Laboratory of Han-D Klenk	N/A
Plasmid: pCAGGs-NP	Laboratory of Han-D Klenk	N/A
Plasmid: pCAGGs-PB1	Laboratory of Han-D Klenk	N/A
Plasmid: pCAGGs-PB2	Laboratory of Han-D Klenk	N/A
Plasmid: pCAGGs-PA	Laboratory of Han-D Klenk	N/A
Plasmid: pRLSV40	Promega	Cat#E2231
Plasmid: pET-19b-DHODH	Zhu et al., 2015a	N/A
Software and Algorithms		
CCP4, version 7.0	CCP4	http://www.ccp4.ac.uk/
BIAevaluation, version 1.1	GE Healthcare	N/A
MicroCal iTC200 Origin Software	GE Healthcare	N/A
Protein Thermal Shift Software version 1.0	Life Technologies	N/A

LEAD CONTACT AND MATERIALS AVAILABILITY

Further information and requests for resources and reagents may be directed to and will be fulfilled by Honglin Li (hlli@ecust.edu.cn), and by the lead contact Ke Xu (xuke03@whu.edu.cn).

EXPERIMENTAL MODEL AND SUBJECT DETAILS

Mice and animal housing

All animals were bred in the specific pathogen-free animal facility and the experiments were approved by the Institutional Animal Care and Use Committee of Institute Pasteur of Shanghai and Wuhan University.

Influenza A virus infection model

We perform all the infection animal experiments following the standard protocols to evaluate influenza pathogenicity (Matsuoka et al., 2009). BALB/c female mice, 6-8 weeks old, were purchased from LingChang Company. Mice were anesthetized using diethyl ether and intranasally challenged with virus suspension, i.e., 4000 or 2000 PFU of A/WSN/33 H1N1 in 50 μL of PBS, 300 PFU of A/SC/09 (H1N1) in 30 μL of PBS and 5×10^4 PFU of NAmut^{H275Y} in 30 μL of PBS, respectively. Diluted compounds were given by intraperitoneal (i.p.) injection once a day. The drug treatment was initiated on day 0 before-infection or days 3, 5, 6, 7, and 8 post-infection respectively and continued for several days.

Zika virus infection model

AG6 mice (mixed gender), 6-8 weeks old, were housed in Institute Pasteur of Shanghai (kindly shared by Prof. Qibin Leng). Mice were intraperitoneal (i.p.)

injected withPBS, S416 (1 mg/kg) and S416 (10 mg/kg) respectively around 3 h before infection, then intraperitoneal infected with 1 × 10^5 PFU of Zika virus SZ-WIV01 in 50 μL of PBS. Compounds were continuous given once per day from day 0 to day 10.

All animal weight and survival were monitored daily, and mice were euthanized until the end of the experiment or when body-weight lost is more than 25%.

METHOD DETAILS

Discovery of DHODHi

To identify novel, potent and low-toxicity DHODHi, we previously conducted a hierarchal structure-based virtual screening against the commercial database that contains ~280,000 compounds towards the ubiquinone-binding site of DHODH, and 18 DHODHi were discovered with IC_{50} values ranging from 0.11 to 18.8 μmol/L among the 47 selected compounds (Diao et al., 2012). After determination of the immunosuppressive activities using the mouse spleen cell proliferation assay and the two-way mixed lymphocyte reaction (MLR) assay, 5 compounds presented anti-proliferation activities towards the activated B cells and T cells with the IC_{50} values lower than 15 μmol/L (Diao et al., 2012). We obtained the co-crystal structure of DHODH with S312 (PDB ID: 4LS1) and S416 (PDB ID: 6M2B, Table S1), two representative and potent inhibitors with IC_{50} values of 29.2 nmol/L and 7.5 nmol/L through structural modification (Li et al., 2015; Zhu et al., 2015a), which are > 10-folds more potent than the active metabolite Teriflunomide (IC_{50} of 307.1 nmol/L) (Discover process shown in **Fig. 1A**). Both S312 and S416 bind at the ubiquinone-binding site, making typical water-bridged hydrogen bond interactions with Q47 and salt bridge interactions with R136 in the inner hydrophilic sub-pocket of DHODH (**Fig. S1A**). The chlorophenyl group connected to the thiazole scaffold of S312 and S416 accommodates the hydrophobic entrance of the binding tunnel. The binding thermodynamic, kinetic studies of S312 and S416, the co-crystallization of S416+DHODH, the kinase-inhibition assays of S312 and S416, the pharmacokinetics study of S416, and the acute toxicity study of S312 were conducted in this study.

Isothermal titration calorimetry (ITC)

ITC measurements were carried out at 25 ℃ on a MicroCal iTC200 (GE Healthcare). For the titration of an inhibitor to DHODH, both were diluted using the buffer (20 mM HEPES, pH 8.0, 150 mM KCl, 10% Glycerol and 10% DMSO). The concentration of DHODH in the cell was 20 μmol/L, and the concentration of inhibitor in the syringe was 150 μmol/L for S312 or 100 μmol/L for S416. All titration experiments were performed by adding the inhibitor in steps of 2 μL. The data were analyzed using Microcal origin software by fitting to a one-site binding model.

Surface plasmon resonance (SPR)

Surface plasmon resonance experiments were performed with a BIAcore T200 (GE Healthcare) according to our previous work (Song et al., 2016). The running buffer

contained 1.05 × PBS, 0.005% (vol/vol) surfactant P20, pH 7.4, and 1% DMSO. The purified DHODH, which was diluted in sodium acetate solution (pH 5.5) with a final concentration of 30 μg/mL, was immobilized on a CM5 sensor chip by amine coupling. All analyte measurements were performed at a flow rate of 30 μL/min. The analyte was diluted in the running buffer from the top concentration. Data processing and analysis were performed using BIAevaluation 1.1 software.

Crystallization and structure determination

The purified DHODH was concentrated to 20 mg/mL and then was incubated with 1 mM S416 and 2 mM DHO for 2 h. Co-crystallization of DHODH with S416 was performed at 20 ^{o}C using the vapor diffusion method as previously described (Li et al., 2015; Zhu et al., 2015a). X-ray diffraction data were collected at 100 K on beamline BL17U1 at the Shanghai Synchrotron Radiation Facility (SSRF). The raw data were processed using the MOSFLM and SCALA programs from the CCP4 suite (Collaborative, 1994; Evans, 2006). The complex structure of DHODH-S416 was determined by molecular replacement using PDB entry 4LS1 (without ligands and water molecules) as the search model. Structure refinement was conducted with REFMAC5 (Murshudov et al., 2011). Coot was used for the interpretation of electron density maps and model building (Emsley et al., 2010). Data collection and refinement statistics were listed in Table S1.

Kinase-inhibition assays

The potential of S312 or S416 as a kinase inhibitor was determined at the concentration of 1 μmol/L against a panel of more than 180 kinases through the commercially available service provided by ICE bioscience.

***In vivo* drug toxicity study**

ICR male mice, 6-8 weeks old, were randomly assigned into five groups with one vehicle group and the other four groups orally administered with S312 at 50, 100, 500, and 1000 mg/kg (n = 4), respectively. Single-dose of S312 was administered on the first day (day 0) of the experiment. During the experiment, all animals were clinically observed every day for 15 days for toxic signs. Body weight and food intake were recorded every two days. At the end of the experiment, all surviving animals were perfused, and tissue samples of heart, liver, lung, and kidney were collected. The tissues were then fixed in 4% paraformaldehyde, dehydrated with sucrose solution, and embedded at optimum cutting temperature. Frozen tissues sections (10 μm) were examined under a Nikon optical microscope at 100X magnification with standard hematoxylin and eosin staining.

Cell cultures, viral strains, and drugs

MDCK, A549, Vero E6, and were obtained from the American Type Culture Collection (ATCC). Huh7 and 293FT cells were kindly provided by Dimitri Lavillette (Institute Pasteur of Shanghai, China). BSR T7/5 cells stably expressing the T7 RNA polymerase gene were kindly provided by Gang Zou (Institute Pasteur of Shanghai,

China). MDCK, A549, Vero E6, Huh7, and 293FT cells were cultured in DMEM (Gibco) supplemented with 10% FBS (Gibco) and 1% P/S (Gibco). BSR T7/5 cells were cultured in GMEM (Gibco) supplemented with 10%FBS (Gibco), 1% L-Glutamine (Gibco), 2% MAA and 1% P/S (Gibco). The A/WSN/33 virus was generated by reverse genetics as previously described (Han et al., 2014). The Oseltamivir-resistant NA^{H275Y} mutant virus (in WSN backbone) was generated by one step site-directed mutagenesis base on reverse genetics as previously described (Han et al., 2014). The A/Sichuan/1/2009(H1N1)(SC09), A/Donghu/312/2006(H3N2), A/Guangzhou/333/99(H9N2) were kindly provided by Prof. Yuelong Shu (Chinese National Influenza Center). Zika virus SZ-WIV01 (KU963796) was kindly provided by Bo Zhang from Wuhan Virology Institute of CAS (Deng et al., 2016). DHODHi S312 and S416 were synthesized using our previously reported synthetic routes (Li et al., 2015; Song et al., 2016). Leflunomide, Teriflunomide, Brequinar, Oseltamivir, Adenosine, Uridine, Cytidine, Guanosine, Orotic acid, Dihydroorotate were purchased from Sigma-Aldrich (St. Louis, MO). Favipiravir was purchased from Selleck.

DHODH knock-out engineered A549 cell line was generated by CRISPR/Cas9 gene-editing system. The following four group primers of sgRNAs were synthesized by Generay Company (Shanghai, China), and cloned into lentiCRISPRv2 vector. 293FT cells were then co-transfected with lentivirus packaging plasmids (lenti-CRISPR v2-DHODH-sgRNA, psPAX2, and PMD2.G) by Lipofectamine® 2000 (ThermoFisher) for 48 h. Three lentivirus packaging plasmids were provided by Dimitri Lavillette (Institute Pasteur of Shanghai, China). The lentivirus supernatants were harvested (mix supernatants of four sgRNAs) after centrifugation and filtration to transduce 1.5×10^6 A549 cells into a 6-well plate for 72 h at 37 ℃. Cells were expanded into 10 mm plates and grown in the presence of puromycin (5 μg/mL) for 3–5 days to select sgRNA-positive cells. Thereafter, single-cell sorting was performed in a 96-well plate to expand single clones, and western blot using the anti-DHODH antibody (Santa Cruz Biotechnology) was performed to detect the expression level of DHODH. The best KO cell clone with the lowest residue DHODH level was finally used. The primers of DHODH sgRNAs were listed in Table S4.

Anti-influenza virus activity

Based on CellTiter-Glo® Cell Viability Assay (Promega), anti-influenza virus activities of the compounds were evaluated. For compound toxicity assays (CC_{50}), MDCK in a 96-well plate at a density of 2×10^4cell/well were cultured in the present of serial dilutions of compounds in infection solution (DMEM + 0.2% BSA + 25 mM HEPES + 1 μg/mL TPCK). After 72 h of incubation in a 37 ℃, 5% CO_2 incubator, 25 μL of CellTiter-Glo® reagent was added to each well, and then the fluorescence (Luminescence) value to reflect cell viability was recorded. For compound effective assays (EC_{50}), the same numbers of cells were seeded in a 96-well plate and each well was infected by 20 $TCID_{50}$ viruses suspended in 50 μL medium together with compounds diluted in another 50 μL medium to reach a final 100 μL culture volume

per well. The cells were then incubated at 37 ℃ until the virus control group reached a 75%-100% cytopathic effect. Cell viability was quantified as above. Data were processed with Graphpad prism software to calculate EC_{50} and CC_{50} values of the compounds.

Detection of anti-ZIKV potency by real-time quantitative PCR

Aliquots of Huh7 were seeded into 12-well plates and infected with the Zika virus (MOI = 0.05). At 4 h post-infection, the medium was removed, and cells were treated with appropriate concentration of compounds in infection medium (DMEM + 2% FBS + 25 mM HEPES). After 7 days, the supernatant was collected and RNA was extracted by TRIzol Reagent (Invitrogen) and subjected to reverse transcription-quantitative PCR with the PrimeScript RT reagent kit (TaKaRa). Then cDNA was quantified with a standard curve generated by determination of copy numbers from serial dilutions of standard Zika cDNA. The relative viral RNA copy numbers were normalized to the vehicle (DMSO) treated sample to calculate inhibition ratio and EC_{50} values. The primers to detect Zika-specific cDNA were listed in Table S4. Data were processed with Graphpad prism software to calculate EC_{50} values of the compounds. The cytotoxicity of the tested drugs on cells was determined by CellTiter-Glo® Cell Viability Assay (Promega). Data were processed with Graphpad prism software to calculate EC_{50} and CC_{50} values of the compounds.

Bright-Glo Pharmacodynamic assay for Ebola Replicon

Ebola virus must be operated in the BSL-4 laboratory. To reduce the biological safety risks, the Ebola virus replicon system was chosen for antiviral activity assay (Jasenosky et al., 2010). BSR T7/5 cells (T7 RNA polymerase stable expressing cell) were seeded in 96-well plates and co-transfected with p3E5E-Luc (25 ng), pCEZ-NP (25 ng), pCEZ-VP35 (25 ng), pCEZ-VP30 (15 ng), and pCEZ-L (150 ng) per well. Afterward, 50 μL of drug dilutions were added into each well and cultured in at 37°C, 5% CO_2 for 24 h. Replicon efficacy was determined by adding 50 μL/well of Bright-Glo reagent to record the fluorescence (Luminescence) values. Data were processed with Graphpad prism software to calculate EC_{50} values of the compounds. The cytotoxicity of the tested drugs on cells was determined by CellTiter-Glo® Cell Viability Assay (Promega). Data were processed with Graphpad prism software to calculate EC_{50} and CC_{50} values of the compounds.

Detection of anti-SARS-CoV-2 potency by real-time quantitative PCR

Vero E6 cells seeded on 48-well plates were infected with virus 2019BetaCoV/Wuhan/WIV04/2019 isolated by Wuhan Institute of Virology, Chinese Academy of Sciences at MOI of 0.03. At the same time, different concentrations of the drugs were added for co-culture, and DMSO dilution was used as a negative control. Cell supernatants were harvest 24 h post-infection and RNA was extracted by a viral RNA extraction kit (Takara, Cat no. 9766). The number of viral copies was detected by real-time quantitative reverse transcription PCR (qRT-PCR) (Takara Cat no.RR820A). Then cDNA was quantified with a standard curve generated by

determination of copy numbers from serial dilutions of the standard SARS-CoV-2 RBD-cloning plasmid. The relative viral RNA copy numbers were normalized to the vehicle (DMSO) treated sample to calculate inhibition ratio and EC50 values. The primers to detect SARS-CoV-2-specific cDNA were listed in Table S4. Data were processed by Graphpad prism software to calculate EC_{50} of the compound. The cytotoxicity of the tested drugs on cells was determined by CCK8 assays (Beyotime, China). Data were processed with Graphpad prism software to calculate EC_{50} and CC_{50} values of the compounds.

IFA assay of SARS-CoV-2

To detect viral protein expression in Vero E6 cells, cells were fixed with 4% paraformaldehyde and permeabilized with 0.5% Triton X-100. The cells were then incubated with the primary antibody (a polyclonal antibody against the NP of a bat SARS-related CoV) after blocking, followed by incubation with the secondary antibody (Alexa 488-labeled goat anti-rabbit, Abcam).

Virus growth curve and plaque assay

To determine the viral growth curve, A549 (WT and $DHODH^{-/-}$) cells were infected with the indicated viruses at an MOI of 0.01. The supernatant of infected cells was collected at different time points post-infection and titrated by plaque assay on MDCK cells. The viral plaque assay with immunostaining was performed as previously described (Matrosovich et al., 2006). Briefly, MDCK cells cultivated in 24-well plates were infected with WSN virus. After 0.5 h of incubation at 37℃ in 5% CO_2, the virus medium was removed, and 1 mL of 2.4% Avicel (FMC Corporation) medium in the presence of indication drugs was added. Cells were incubated for another 48 h at 37℃ in 5% CO_2 and then fixed with 4% PFA. Immunostaining was performed using an anti-NP polyclonal antibody conjugated with HRP (Antibody Research Center, Shanghai Institute of Biological Science). True Blue substrate (KPL) was added to visualize the plaques.

Influenza mini-replicon system and DHODH substrates assay

The 293FT cells were seeded into 24-well plates and were transfected with influenza A/WSN/33 virus PB1-, PB2-, PA- and NP- expressing plasmids (100 ng each), the influenza virus-specific RNA polymerase I driven firefly luciferase reporter (pPoⅡ-NP-luc) (100 ng, provide by Han-D Klenk, Marburg University), and the Renilla luciferase reporter pRLSV40 10 ng, Promega). After 12 h, the transfected 292FT cells were treated with different concentrations of the test compounds or exogenous nucleosides (Adenosine, Uridine, Cytidine, Guanosine) or orotic acid or dihydroorotate. Cells were harvested after another 24 h and firefly luciferase and Renilla luciferase expression were determined using the Dual-Luciferase Assay Kit (Promega).

Cytokine and chemokine measurements

BALB/c female mice, 6 - 8 weeks old, were anesthetized and intranasally challenged

with 2000 PFU of A/WSN/33 H1N1 in 50 μL of PBS at day 0. Afterward, mice were weighed daily and administrated daily by intraperitoneal injection until the body weight dropped to about 80%. At the end of the experiment, mice were euthanized and the bronchoalveolar lavage fluid was taken as previously published (Au - Van Hoecke et al., 2017). The samples were frozen at −80°C for subsequent analysis. The samples of lavage fluid were centrifuged at 3000 g for 10 min at 4 ℃ and the cytokines and chemokines levels were measured using the U-PLEX Biomarker Group 1 (ms) assays. All assays were performed according to manufacturer's instructions, tests were performed in duplicates, and without alterations to the recommended standard curve dilutions.

QUANTIFICATION AND STATISTICAL ANALYSIS

The EC_{50} and CC_{50} values of the compounds are calculated by the nonlinear fitting equation in GraphPad Prism 8. One-way or two-way analysis of variance (ANOVA) was used to test for statistical significance. The log-rank test was used for survival curves analyses. Only p values of 0.05 or lower were considered statistically significant (NS (not significant), $p > 0.05$; *,# $p < 0.05$; **,## $p < 0.01$; ***,### $p < 0.001$.). For statistical analysis, the GraphPad Prism 8 software package (GraphPad Software) or OriginPro 2020 SR1 (9.7.0.188) was used.

DATA AND CODE AVAILABILITY

The protein structure data has been uploaded to the Protein Data Bank with accession number 6M2B.

SUPPLEMENTAL FIG. LEGENDS

Fig. S1. Binding mode of S416, and selectivity profiles of S312 and S416 against a panel of more than 180 kinases. Related to Fig. 1. (A) The binding mode of S416 to DHODH in the X-ray complex crystal structure (PDB ID: 6M2B). Key residues are shown as thin gray sticks. Hydrogen bonds are displayed as black dashed lines. Water molecules are depicted as small red balls. **(B)** and **(C)** The inhibitory activity of S312 and S416 against the kinases were determined at 1 μmol/L. The kinome tree illustrations were generated by KinMap (http://www.kinhub.org/kinmap/). Kinases with inhibition rate < 45% were labeled with green dots while kinases with the inhibition rate of 50-55% were labeled with red dots.

Fig. S2. The acute toxicity study of S312. **Related to Fig. 1. (A)** Body weight changes of the mice orally treated with S312 (50, 100, 500, and 1000 mg/kg) in the acute toxicity study. No significant body weight loss was observed as compared to control even with the maximum tolerated dose of S312 (1000 mg/kg). The results were presented as means ± SEM. Statistical analysis: two-way ANOVA. NS, $p > 0.05$. **(B)** Hematoxylin and eosin staining of various tissues under an optical microscope for histopathological morphology analysis at 14 days after oral administration of 1000 mg/kg S312 in ICR mice. No abnormalities were observed.

Fig. S3. Broad-spectrum antiviral activities of DHODHi. Related to Fig.s 1 and 2. (A) Anti-Ebola replication potency. BSR-T7/5 cells were transfected with the EBOV mini-genome replication system (NP, VP35, VP30, MG, and L) in the presence of increasing concentrations of Teriflunomide, Brequinar, S312, and S416 respectively. Inhibitory effects of these compounds (EC_{50}) to EBOV mini-genome replication were determined using Bright-Glo Luciferase Assay. CC_{50} of compounds were determined by analyzing BSR-T7/5 cell viability using CellTiterGlo Assay. The results are presented as a mean of at least two replicates ± SD. **(B)** Anti-Zika virus potency. Huh7 cells were infected with Zika virus (MOI=0.05) for 4 h and then treated with increasing concentrations of compounds Teriflunomide, Brequinar, S312, and S416, respectively. The viral yields in cell supernatants were then quantified by qRT-PCR to reflect the replication efficiency of Zika virus. **(C)** Anti-SARS-CoV-2 virus potency at MOI = 0.03. Aliquots of Vero E6 cells were seeded in 96-well plates and then infected with Beta CoV/Wuhan/WIV04/2019 at MOI of 0.03. At the same time, different concentrations of the compounds were added for co-culture. Cell supernatants were harvested 48 h.p.i. and RNA was extracted and quantified by qRT-PCR to determine the numbers of viral RNA copies. For both B and C, inhibition potencies (EC_{50}) of the compounds were determined by percentage viral RNA reductions as compared to control treatment (DMSO), and their cytoxicities (CC_{50}) were determined using cell viability assay. The results were presented as a mean of at least three replicates ± SD.

Fig. S4. S312 is active against Oseltamivir-resistant strain NAmut^{H275Y}. Related to Fig. 3. (A) Viral growth curve. MDCK cells were infected with NAmut^{H275Y} and

influenza virus A/WSN/33 H1N1 (MOI = 0.001). The supernatant of infected cells was collected at various h post-infection. Viral titers were determined by plaque assay on MDCK cells. **(B, C)** BALB/c mice were intraperitoneally injected with PBS, Oseltamivir (20 mg/kg), and S312 (2.5 mg/kg) respectively around 3 h before infection, and then intranasal infected with $5x10^4$ PFU of $NAmut^{H275Y}$ and 4000 PFU of influenza virus A/WSN/33 H1N1. Compounds were continuous given once per day from D0–D13. Body weight and survival of the mice were monitored for 14 days or when the body weight lost more than 25%, respectively. (B) PBS group n = 2, the other groups n = 3; (C) n = 4 mice per group. The dotted lines indicate endpoint for mortality (25% weight loss) separately. The results were presented as mean ± SD for (A) and mean ± SEM for weight cruves. Pound signs (#) indicate significance between $NAmut^{H275Y}$+Osel (20 mg/kg) and Wild type+Osel (20 mg/kg). #, $p < 0.05$; ##, $p < 0.01$; and , $p < 0.001$. Statistical analysis, two-way ANOVA for weight cruves and the log-rank test for survival cruves.

Fig. S5. Experimental procedure for DHODH knockout in A549 cells. Related to Fig. 4. (A) Lentivirus-packaged sgRNA against DHODH was transduced into A549 cells and selected by puromycin with a concentration of 5 ug/mL. Subsequently, single-cell sorting was performed in a 96-well plate to obtain single clones. The best KO cell clone with the lowest residue DHODH level was finally used. **(B)** Cell growth curve. A549 cells and $DHODH^{-/-}$ cells were respectively seeded in 48 well plates with 1×10^5 cells/well. Cells were then counted by hemocytometer at each time points of 2 h，24 h, 48 h, and 72 h. The results were presented as means ± SD. Statistical analysis, two-way ANOVA. NS, $p > 0.05$.

Fig. S6. Cytokine and chemokine measurements. Related to Fig. 5. (A) BALB/c mice were intranasally infected with 2000 PFU of influenza virus A/WSN/33 H1N1. Then, mice were subjected to intraperitoneal injection (i.p.) with Oseltamivir (20 mg/kg), S312 + Oseltamivir (5 mg/kg + 20 mg/kg) once a day from D8–D13. The green bar indicates the period of drug administration. Body weight loss and survival of the mice were monitored for 14 days or until weight change to 25%. Osel (20 mg/kg) group n = 4, the other groups n = 5. The dotted lines indicate initial weight and endpoint for mortality (25% weight loss). The results were presented as means ± SEM for weight curves. Asterisks (*) indicate significance when the indicated group compared with the non-treatment group. ** $p < 0.01$. Statistical analysis, two-way ANOVA for weight curves and the log-rank test for survival curves. $p > 0.05$, unlabeled. **(B)** The cytokines and chemokines were measured by Meso Scale Discovery (MSD). The data were expressed as mean +SD and shown as bar charts. The statistical analyses were performed using one-way ANOVA followed by Turkey post-hoc test. The plot function, ANOVA, and the post-hoc functions were provided by OriginPro 2020 SR1 (9.7.0.188). $p < 0.05$ was considered statistically significant and therefore the "significance level" parameters of the above functions were set to 0.05.

Fig. S7. Drug efficacy of S416 in Zika-infected AG6 mice. Related to Fig.s 3 and 5. (A) AG6 mice (mixed gender) were intraperitoneal injected (i.p.) with PBS, S416 (1 mg/kg) and S416 (10 mg/kg) respectively around 3 h before infection, and intraperitoneal infected with 10^5 PFU of Zika virus. Compounds were continuous given once per day from D0–D10. The body weight loss and survival were monitored for 14 days or until body weight lost more than 25% (PBS group n = 3, the other groups n = 4). The dotted lines indicate initial weight and endpoint for mortality (25% weight loss). The body weights are present as the mean percentage of weight change ±SEM and survival curves were shown. Body weights and survival curves of male **(B)** and female **(C)** AG6 mice in A were shown respectively. Statistical analysis, two-way ANOVA for weight curves, and the log-rank test for survival curves. $p > 0.05$, unlabeled.

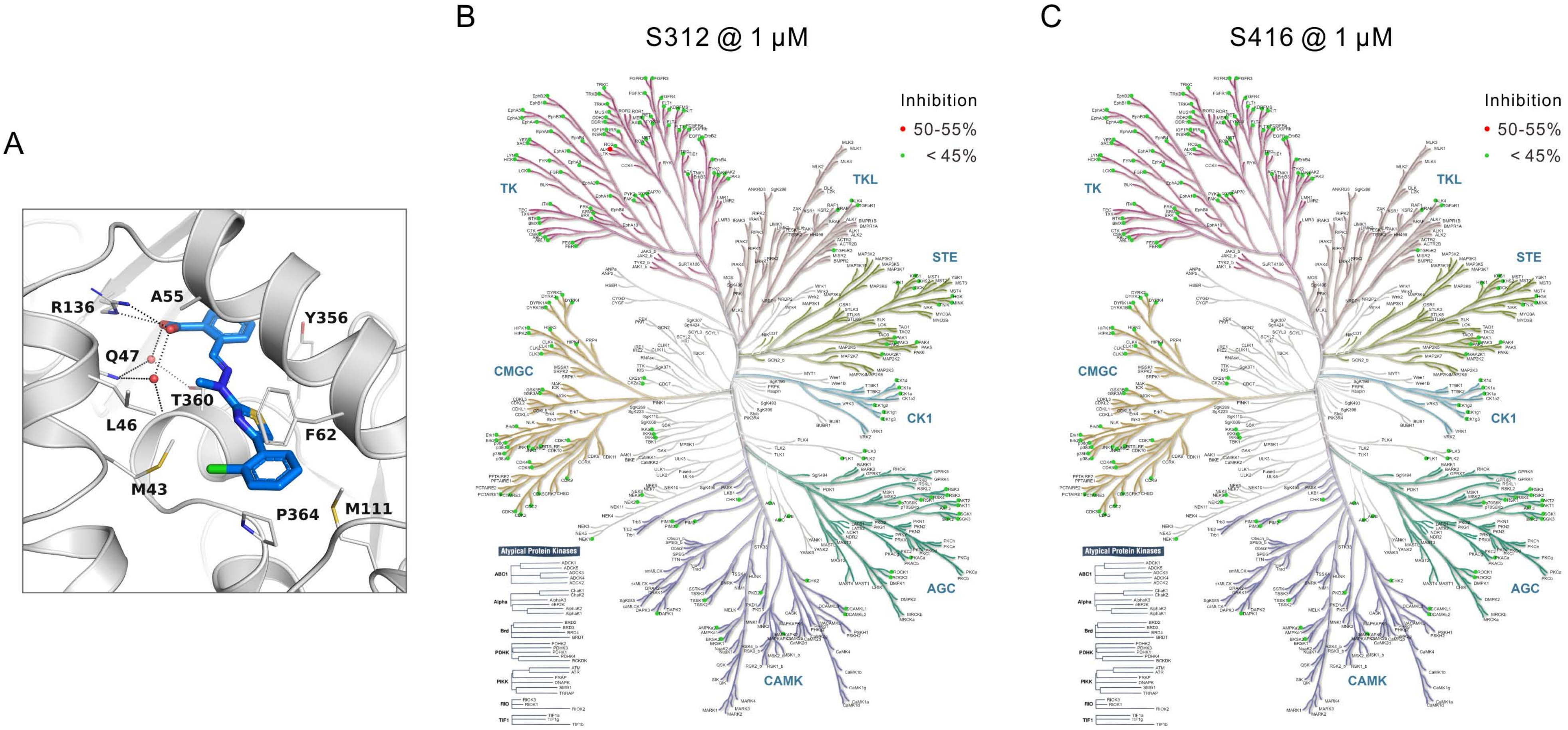
A
R136
A55
Y356
Q47
T360
L46
F62
M43
P364
M111
B
S312 @ 1 µM
C
S416 @ 1 µM
Inhibition
50-55%
< 45%
TK
TKL
STE
CMGC
CK1
AGC
CAMK
Atypical Protein Kinases
ABC1
Alpha
Brd
PDHK
PIKK
RIO
TIF1

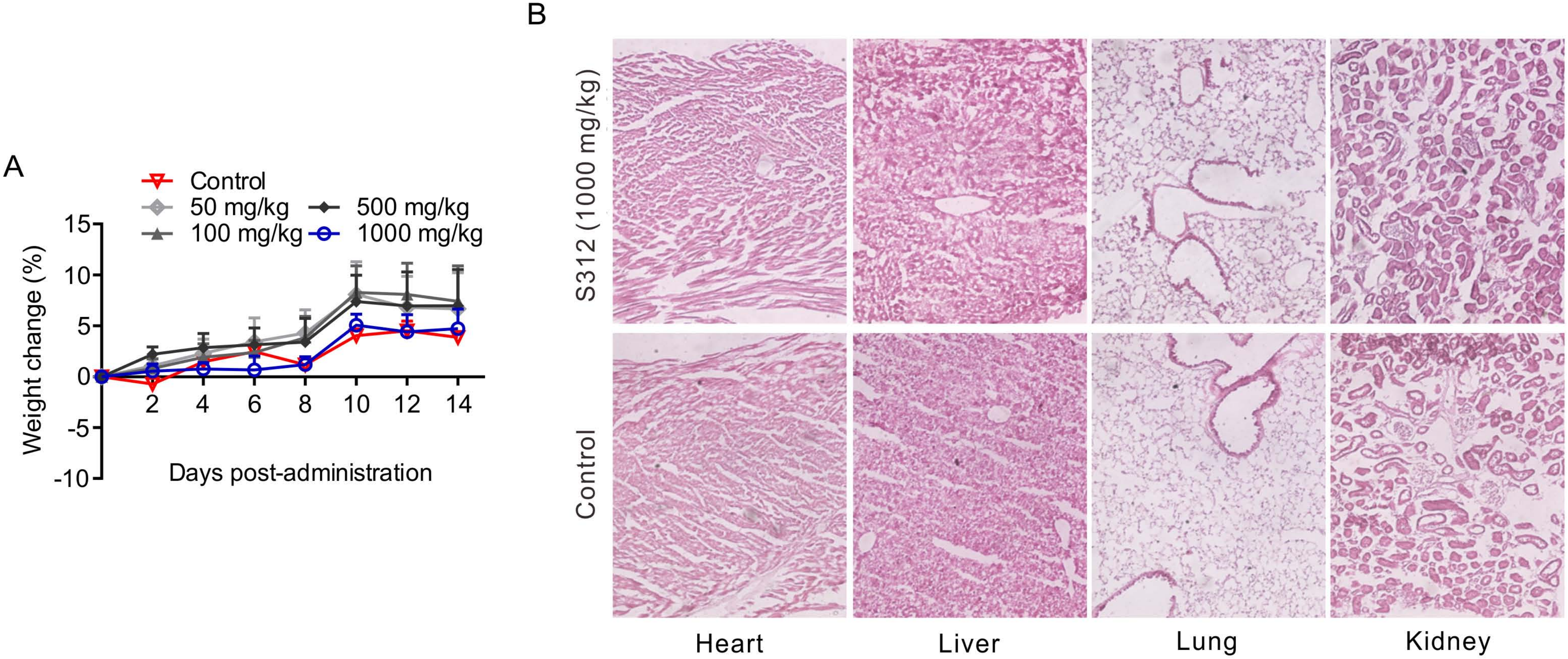
A
Control
50 mg/kg
100 mg/kg
500 mg/kg
1000 mg/kg
Weight change (%)
Days post-administration
B
S312 (1000 mg/kg)
Control
Heart
Liver
Lung
Kidney

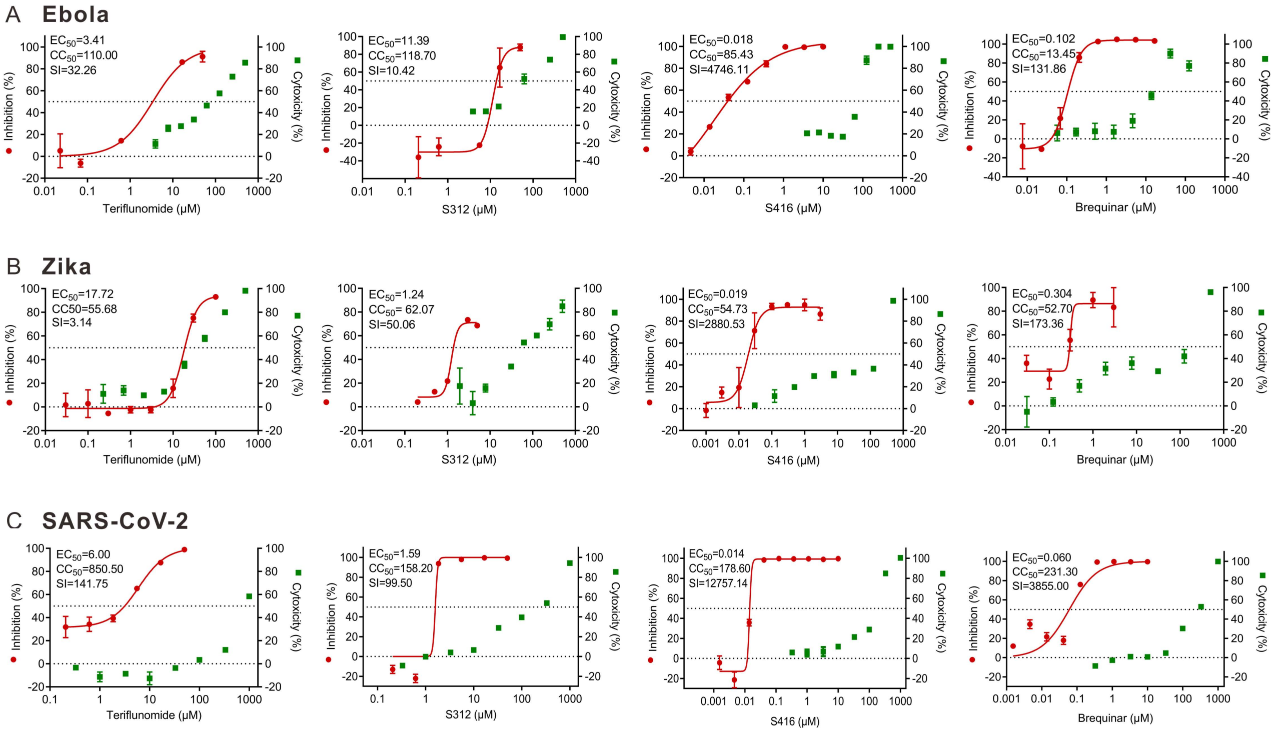

A Ebola
EC50=3.41
CC50=110.00
SI=32.26
Teriflunomide (μM)
EC50=11.39
CC50=118.70
SI=10.42
S312 (μM)
EC50=0.018
CC50=85.43
SI=4746.11
S416 (μM)
EC50=0.102
CC50=13.45
SI=131.86
Brequinar (μM)
B Zika
EC50=17.72
CC50=55.68
SI=3.14
Teriflunomide (μM)
EC50=1.24
CC50= 62.07
SI=50.06
S312 (μM)
EC50=0.019
CC50=54.73
SI=2880.53
S416 (μM)
EC50=0.304
CC50=52.70
SI=173.36
Brequinar (μM)
C SARS-CoV-2
EC50=6.00
CC50=850.50
SI=141.75
Teriflunomide (μM)
EC50=1.59
CC50=158.20
SI=99.50
S312 (μM)
EC50=0.014
CC50=178.60
SI=12757.14
S416 (μM)
EC50=0.060
CC50=231.30
SI=3855.00
Brequinar (μM)
Inhibition (%)
Cytoxicity (%)

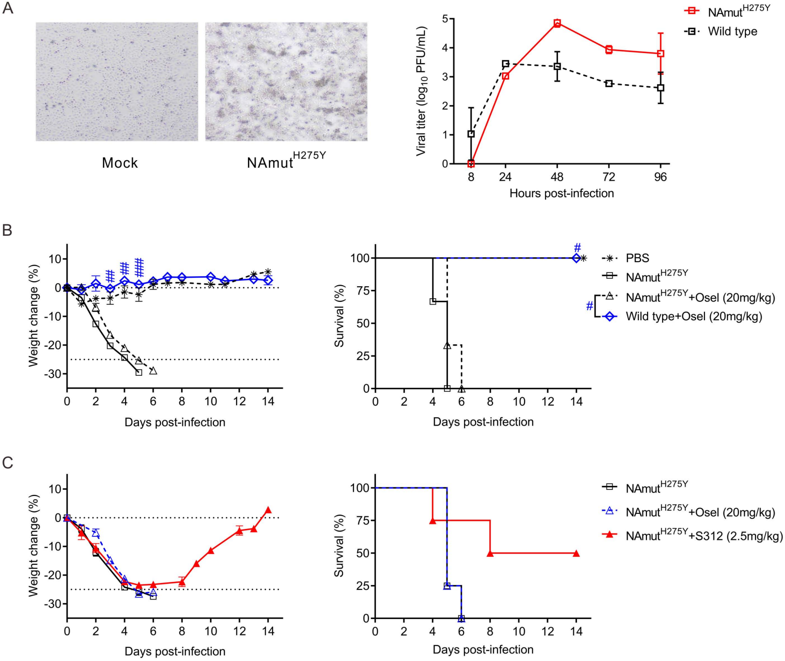

A
Mock
NAmutH275Y
Viral titer (log10 PFU/mL)
Hours post-infection
NAmutH275Y
Wild type
B
Weight change (%)
Days post-infection
Survival (%)
Days post-infection
PBS
NAmutH275Y
NAmutH275Y+Osel (20mg/kg)
Wild type+Osel (20mg/kg)
#
C
Weight change (%)
Days post-infection
Survival (%)
Days post-infection
NAmutH275Y
NAmutH275Y+Osel (20mg/kg)
NAmutH275Y+S312 (2.5mg/kg)

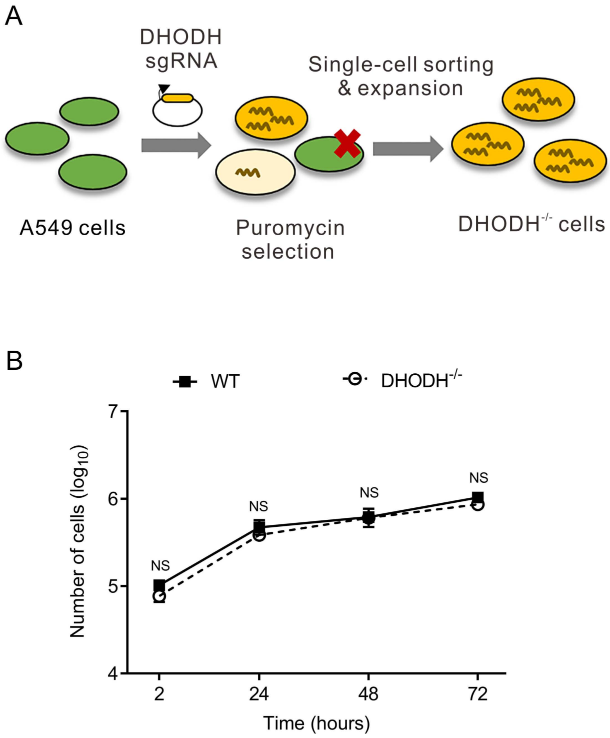
A
DHODH sgRNA
Single-cell sorting & expansion
A549 cells
Puromycin selection
DHODH-/- cells
B
WT
DHODH-/-
Number of cells (log10)
7
6
5
4
NS
NS
NS
NS
2
24
48
72
Time (hours)

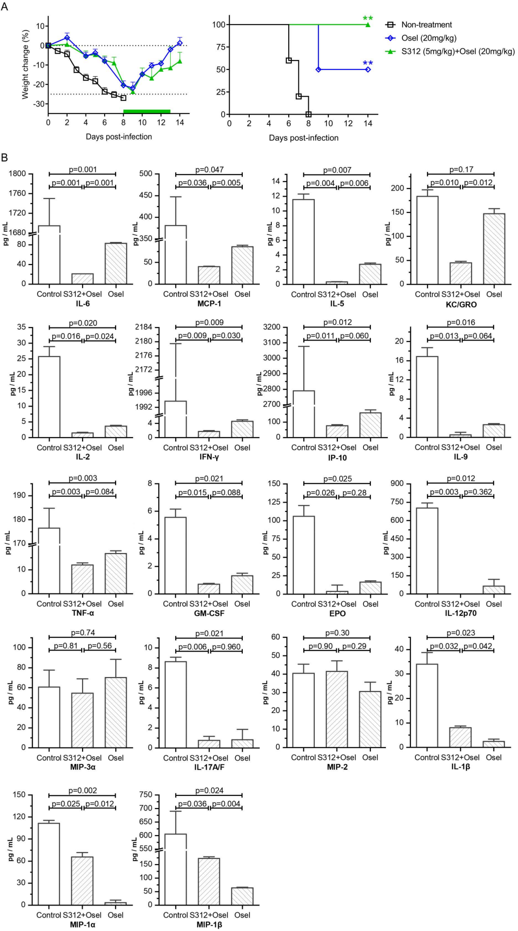
A
Weight change (%)
Days post-infection
Non-treatment
Osel (20mg/kg)
S312 (5mg/kg)+Osel (20mg/kg)
**
B
pg / mL
Control S312+Osel Osel
IL-6
MCP-1
IL-5
KC/GRO
IL-2
IFN-γ
IP-10
IL-9
TNF-α
GM-CSF
EPO
IL-12p70
MIP-3α
IL-17A/F
MIP-2
IL-1β
MIP-1α
MIP-1β
p=0.001 p=0.001 p=0.001
p=0.047 p=0.036 p=0.005
p=0.007 p=0.004 p=0.006
p=0.17 p=0.010 p=0.012
p=0.020 p=0.016 p=0.024
p=0.009 p=0.009 p=0.030
p=0.012 p=0.011 p=0.060
p=0.016 p=0.013 p=0.064
p=0.003 p=0.003 p=0.084
p=0.021 p=0.015 p=0.088
p=0.025 p=0.026 p=0.28
p=0.012 p=0.003 p=0.362
p=0.74 p=0.81 p=0.56
p=0.021 p=0.006 p=0.960
p=0.30 p=0.90 p=0.29
p=0.023 p=0.032 p=0.042
p=0.002 p=0.025 p=0.012
p=0.024 p=0.036 p=0.004

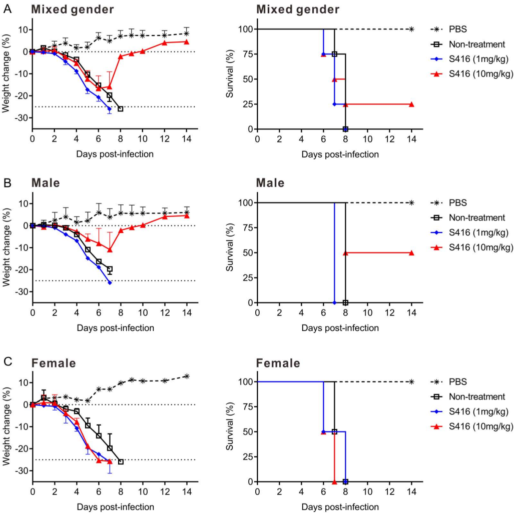
A
Mixed gender
Weight change (%)
Days post-infection
Survival (%)
PBS
Non-treatment
S416 (1mg/kg)
S416 (10mg/kg)
B
Male
C
Female

Table S1. Data collection and refinement statistics for the DHODH-S416 co-crystal structure. Related to Figure 1.

**Data collection**	
Wavelength (Å)	0.97852
Space group	$P3_221$
Cell dimensions	
a, *b*, *c* (Å)	91.04, 91.04, 122.18
α, β, γ (°)	90.00, 90.00, 120.00
Resolution (Å)	48.34-1.76 (1.86-1.76)
R_{merge} (%)	10.7 (44.2)
$I / \sigma(I)$	15.0 (5.4)
Completeness (%)	100 (100)
Redundancy	10.3 (9.9)
**Refinement**	
Resolution (Å)	48.34-1.76 (1.86-1.76)
No. of reflections used in refinement	55638
R_{work} / $R_{free(\%)}$	16.7/19.3
Total atoms	3059
Protein atoms	2847
Ligand (FMN/ORO/S416) atoms	67
Water	145
Wilson B-factor (Å^2)	16.6
Average B, all atoms (Å^2)	20.0
R.m.s. deviations	
Bond lengths (Å)	0.010
Bond angles (°)	1.742
Ramachandran plot	
Preferred regions (%)	96.31
Allowed regions (%)	2.84
Outliers (%)	0.85
**PDB code**	6M2B

*Values in parentheses are for the highest-resolution shell.

Table S2. Binding properties of DHODHi. Related to Figure 1.

Compound	ITC				SPR		
	ΔH (kJ/mol)	$-T\Delta S$ (kJ/mol)	ΔG (kJ/mol)	K_D (M)	k_{on} ($M^{-1}s^{-1}$)	k_{off} (s^{-1})	K_D (M)
**S312**	-38.83	-6.23	-45.06	1.29×10^{-8}	3.12×10^{5}	6.33×10^{-3}	2.03×10^{-8}
**S416**	-33.51	-13.22	-46.74	6.41×10^{-9}	1.76×10^{6}	2.97×10^{-3}	1.69×10^{-9}

Table S3. Pharmacokinetics profiles of DHODHi S312 and S416. Related to Figure 1.

**Parameter**[a]	**S312 (Li et al., 2015)**	**S416**
IV dose (mg/kg)	1	1
C_0 (μg/L)	34478.30 ±18771.00	6709.82 ±188.11
$AUC_{0-\infty}$ (μg*h/L)	23318.81 ±4501.24	47558.65 ±6150.08
Cl (L/h/kg)	0.04 ±0.01	0.02 ±0.00
Vd_{ss} (L/kg)	0.35 ±0.02	0.14 ±0.02
PO dose (mg/kg)	10	10
$T_{1/2}$ (h)	8.20 ±1.72	9.12 ±2.91
$AUC_{0-\infty}$ (μg*h/L)	53047.73 ±11030.40	362768.43 ±132144.96
C_{max} (μg/L)	5310.50 ±1910.47	24379.63 ±7533.42
T_{max} (h)	1.00 ±0.87	4.00 ±0.00
F (%)	22.75 ±4.73	76.28 ±27.79

[a] Compounds were dosed to an equal number of male Sprague-Dawley rats by intravenous (IV) and oral (PO) administration respectively (*n* = 3).

Table S4. **Primers used in this paper. Related to the KEY RESOURCES TABLE**

Primer	Sequence (5'-3')
A/WSN/33(H1N1)(NAmut^{H275Y})	ACATTCCTCGTAGTAAGAATTAGGTGCATTCAACTCTATCGA
A/WSN/33(H1N1)(NAmut^{H275Y})	TCGATAGAGTTGAATGCACCTAATTCTTACTACGAGGAATGT
DHODH_sg1 F	CACCGACACCTGAAAAAGCGGGCCC
DHODH_sg1 R	AAACGGGCCCGCTTTTTCAGGTGTC
DHODH_sg2 F	CACCGCGTGGACGGACTTTATAAGA
DHODH_sg2 R	AAACTCTTATAAAGTCCGTCCACGC
DHODH_sg3 F	CACCGCGCAGAAGGGGTGCGCGTAC
DHODH_sg3 R	AAACGTACGCGCACCCCTTCTGCGC
DHODH_sg4 F	CACCGTCAAAGAGTTGGGCATCGAT
DHODH_sg4 R	AAACATCGATGCCCAACTCTTTGAC
Zika virus F	CTCCAGGATGCAAGTCTAAG
Zika virus R	ACCCAGCAGGAACTTCAGGA
SARS-CoV-2 F	CAATGGTTTAACAGGCACAGG
SARS-CoV-2 R	CTCAAGTGTCTGTGGATCACG
